# The Global Landscape of *Plasmodium falciparum* Drug Resistance Markers, 2005–2025: A Systematic Review and Meta-Analysis

**DOI:** 10.3390/pathogens15020179

**Published:** 2026-02-06

**Authors:** Felix Habarugira, Jeanne Batamuriza, Raphael Ndahimana, Jules Ndoli Minega, Mame Massar Dieng, Masceline Jenipher Mutsaka-Makuvaza, Tolessa Muleta Daba, Youssef Idaghdour, Leon Mutesa

**Affiliations:** 1 Department of Biomedical Laboratory Sciences, School of Health Sciences, College of Medicine and Health Sciences, University of Rwanda, Kigali P.O. Box 3286, Rwanda; camaradee@gmail.com (F.H.); batamurizajeanne04@gmail.com (J.B.); 2 Pathology Department, Research Directorate, University Teaching Hospital of Butare, Huye P.O. Box 254, Rwanda; ndolijules@chub.rw; 3 Department of Microbiology and Parasitology, School of Medicine and Pharmacy, College of Medicine and Health Sciences, University of Rwanda, Huye P.O. Box 3286, Rwanda; ndahimana.raphael@gmail.com (R.N.); mascelinejeni@gmail.com (M.J.M.-M.); 4 Program in Biology, Division of Sciences and Mathematics, New York University Abu Dhabi, Abu Dhabi P.O. Box 129188, United Arab Emirates; mame.massar.dieng@nyu.edu (M.M.D.); youssef.idaghdour@nyu.edu (Y.I.); 5 Department of Medical Biochemistry, Molecular Biology and Genetics, School of Medicine and Pharmacy, College of Medicine and Health Sciences, University of Rwanda, Huye P.O. Box 3286, Rwanda; t.daba@ur.ac.rw; 6 Centre for Human Genetics, University of Rwanda, Kigali P.O. Box 4285, Rwanda

**Keywords:** *Plasmodium falciparum*, drug resistance markers, treatment outcome, artemisinin resistance

## Abstract

Malaria remains a global health threat, with *Plasmodium falciparum* causing most deaths, especially in sub-Saharan Africa. Although artemisinin-based therapies reduce the burden, drug-resistant parasites threaten control efforts. Mapping the distribution and evolution of molecular resistance markers is vital for evidence-based strategies. This systematic review mapped the global distribution, pooled prevalence, and temporal trends of key *P. falciparum* antimalarial resistance markers. Following the PRISMA methodology (PROSPERO: CRD4202511098991), databases (PubMed, Web of Science, Scopus, and Google Scholar) and gray sources were searched (July 2005–July 2025). Data were extracted in Rayyan, assessed via the JBI prevalence tool, and analyzed using Python v3.13 for WHO regional distribution, temporal trends, and treatment outcome trends. Of the 1972 records, 261 studies from 64 countries qualified for inclusion in this review. The pooled prevalence was highest for *pfdhfr* (85.7%), followed by *pfcrt* (78.0%), *pfdhps* (73.7%), pfmdr1 (60.5%), and *pfk13* (45.0%). High heterogeneity (I^2^ > 95%) and rising *pfk13* since 2012 highlight emerging artemisinin resistance, while persistent *pfdhfr/pfdhps* mutations show that ongoing sulfadoxine–pyrimethamine (SP) pressure on P. *falciparum* drug resistance, decreased parasite clearance, and treatment failure remain widespread and evolving in Africa. Integrating molecular surveillance into national malaria programs is essential to guide treatment modalities and support progress toward malaria elimination.

## 1. Introduction

Malaria remains a major global public health burden, with approximately 249 million cases reported across 85 endemic countries in 2024 [1]. The African region continues to bear the highest burden, accounting for approximately 94% of all malaria cases [1]. Human malaria is mainly caused by *Plasmodium falciparum* (Welch, 1897), *P. vivax*, *P. malariae*, *P. ovale* (curtisi/wallikeri), and *P. knowlesi* (transmission is predominantly anthroponotic; however, *P. knowlesi* is zoonotic, with non-human primates serving as reservoirs in Southeast Asia). Among the five Plasmodium species known to infect humans, *P. falciparum* is the most lethal, contributing the most to malaria-related morbidity and mortality in endemic foci [2].

Over the past two decades, significant strides have been made in malaria control through vector management, preventive measures, and effective treatment regimens, such as artemisinin-based combination therapies (ACTs) [3]. However, these gains are increasingly threatened by the emergence and spread of *P. falciparum* resistance to several antimalarial drugs, including chloroquine, sulfadoxine–pyrimethamine (SP), mefloquine, and, more recently, artemisinin combination therapies (ACTs) [4]. The continued rise in drug resistance threatens treatment efficacy and hinders progress toward malaria control and elimination [5]. Resistance development is primarily driven by spontaneous mutations or gene duplications in key parasite genes under drug selection pressure [6].

Several genetic markers in *P. falciparum* have been validated for monitoring drug resistance. These include *pf*crt mutations associated with chloroquine resistance [7], ppf plasmepsin 2/3 gene amplifications related to piperaquine resistance [8], pfmdr1 polymorphisms linked to mefloquine and amodiaquine resistance, mutations in dhfr and dhps conferring resistance to SP, and *pf*kelch13 mutations associated with delayed parasite clearance following artemisinin therapy [9]. The distribution and prevalence of these molecular antimalarial drug resistance markers vary by region and time [10]. For instance, chloroquine resistance first emerged independently in South-East Asia and South America before spreading to Africa and other endemic areas [11]. Similarly, artemisinin resistance has recently been documented in East Africa following its initial emergence in the Greater Mekong Subregion [12,13]. *P. falciparum* drug resistance is a serious threat because it reduces treatment effectiveness, and reduced parasite clearance is the primary mechanism that connects drug resistance to treatment outcomes [14].

Despite extensive regional reports on antimalarial resistance markers, the evidence remains fragmented and unevenly distributed across WHO regions. Therefore, a global synthesis is needed to quantify the pooled prevalence, compare geographic patterns, and examine temporal trends to inform surveillance priorities and policy decisions. In contrast to prior reviews that focus on single regions or specific marker families, this review synthesizes five major resistance marker gene groups across all WHO malaria regions over a 20-year period, combining pooled prevalence estimates with temporal analyses to highlight programmatic implications for ACT efficacy and SP-based prevention policies.

This systematic review aimed to (1) map the geographical distribution of key *P. falciparum* drug resistance markers, (2) examine their temporal trends in prevalence, and (3) assess trends in malaria treatment outcomes in relation to the spread of drug resistance. By synthesizing the available evidence, this review will complement the resources to guide surveillance systems, treatment policies, and research priorities.

## 2. Materials and Methods

### 2.1. Protocol Registration

This systematic review and meta-analysis adhered to the PRISMA 2020 guidelines [15]. The protocol was registered in the PROSPERO International Prospective Register of Systematic Reviews (ID = CRD4202511098991).

### 2.2. Literature Search and Eligibility Criteria

A comprehensive literature search was conducted in four databases, including PubMed, Web of Science, Scopus, and Google Scholar; gray literature and manual searching was conducted between August and September 2025 to identify studies reporting the distribution of antimalarial drug resistance markers. A Google Scholar search was performed using the Publish or Perish software 24.6.0 [16,17], and feasibility screening was limited to the first 200 results [18]. Table 1 summarizes the full search strings employed and the number of articles retrieved from each database. In addition, the reference lists of all eligible studies were hand-searched, and gray literature searches were conducted through institutional repositories to identify unpublished and non-indexed studies. Eligible studies included peer-reviewed observational (cross-sectional, cohort, or surveillance) or clinical trial studies reporting molecular markers of *P. falciparum* drug resistance in human samples published in English between 1 July 2005, and 1 July 2025. Studies were excluded if they involved non-human models, lacked data on molecular resistance markers, involved other malaria species rather than *P. falciparum*, or were reviews, commentaries, editorials, or conference abstracts lacking full data.

### 2.3. Study Selection and Data Extraction

**Figure 1:**
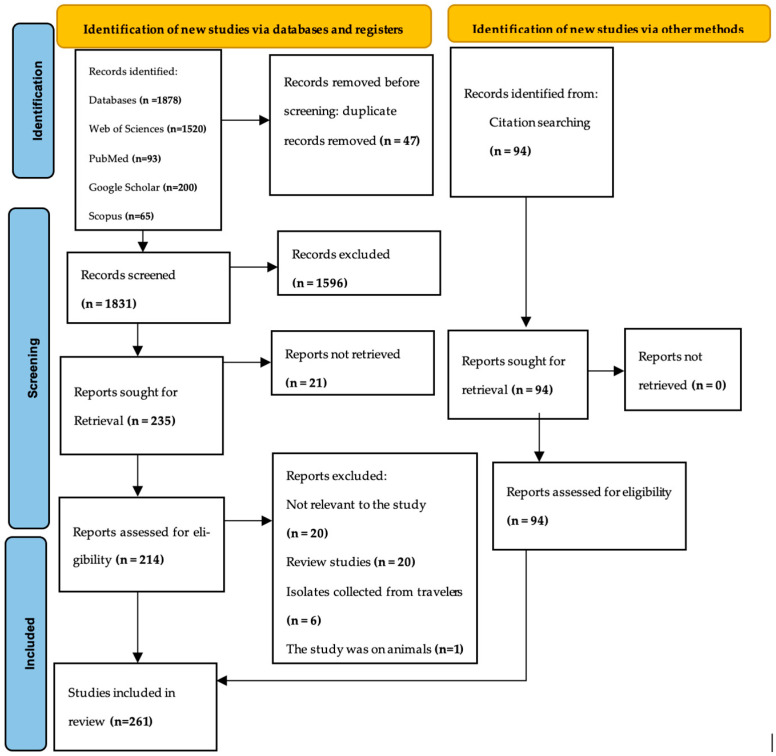
PRISMA flow chart: details of screening procedure.

All retrieved articles were imported into Rayyan software1.4.4 [19] for management and screening. Duplicate articles were automatically removed by the software, and its machine learning conflict-detection tool flagged potential conflicts during the title and abstract review. Two reviewers (BD and TJP) independently screened the titles and abstracts and assessed the full texts of potentially relevant articles. The reasons for exclusion at the full-text assessment stage are recorded in the file of excluded studies with authors (Supplementary File S2). Two reviewers independently extracted the data using a piloted data extraction form. The extracted data included study variables such as publication year, country/region, molecular markers, sample size (tested sample size), prevalence of each key mutation, associated antimalarial drugs, and reported treatment outcomes. Disagreements between the reviewers were resolved through discussion or adjudication by a third reviewer. The final data abstraction file containing authors is available in the Supplementary Files (Supplementary File S3), while the overall process of study identification, screening, and inclusion is summarized in the PRISMA flow diagram (Figure 1), with PRISMA 2020 Checklist (Supplementary File S1).

### 2.4. Quality Assessment of Individual Studies

Potential publication bias was evaluated visually using funnel plots, which are presented in the Supplementary Materials (Figure S1). The methodological quality of included studies was assessed using the Joanna Briggs Institute (JBI) Critical Appraisal Checklist for Prevalence Studies, ensuring the robustness and reliability of pooled estimates. The Supplementary Files contain individual studies’ quality assessments (File S4). 

### 2.5. Data Analysis

Data analysis was conducted using Python version 3.13, primarily employing the statsmodels package for meta-analysis, with pandas for data management and matplotlib for visualization.

### 2.6. Primary Meta-Analysis of Resistance Marker Prevalence

The primary effect measure was the prevalence proportion of each molecular antimalarial resistance marker (*pf*crt, *pf*mdr1, *pf*dhfr, *pf*dhps, and pfk13), calculated as the number of samples positive for a given mutation divided by the total number of successfully genotyped samples in each study. To stabilize variances and address skewness inherent in proportion data, prevalence estimates were transformed using the Freeman–Tukey double arcsine transformation prior to pooling. Pooled prevalence estimates and corresponding 95% confidence intervals (CIs) were calculated using a random-effects meta-analysis, accounting for the expected between-study variability arising from differences in study design, geographic location, population characteristics, and sampling periods. Between-study variance (τ^2^) was estimated using the DerSimonian–Laird method, and statistical heterogeneity among studies was assessed using Cochran’s Q test and quantified with the I^2^ statistic, representing the proportion of total variability attributable to true heterogeneity rather than chance. I^2^ values of approximately 25%, 50%, and 75% were interpreted as low, moderate, and high heterogeneity, respectively. Given the consistently high heterogeneity observed across the resistance markers, the use of a random-effects model was considered appropriate. Subgroup analyses were conducted according to the World Health Organization (WHO) malaria-endemic regions and the study period to explore the geographic and temporal variability in resistance prevalence. Temporal trends were further examined using meta-regression, with the publication year included as a covariate. The following WHO regional classification was applied: (1) African Region (AFRO), 47 countries; (2) South-East Asia Region (SEARO), 9 countries; (3) Western Pacific Region (WPRO), 10 countries; (4) Eastern Mediterranean Region (EMRO), 9 countries; and (5) Region of the Americas (AMRO), 21 countries.

## 3. Results

### 3.1. Study Sites and Study Period

A total of 261 studies published between 2005 and 2025 met the inclusion criteria and were included in the pooled analyses. These studies covered five WHO regions including the African Region (AFRO), which contributed the majority of the studies (n = 166; 63.6%), followed by the South-East Asia Region (SEARO; n = 55; 21.1%), Western Pacific Region (WPRO; n = 16; 6.1%), Eastern Mediterranean Region (EMRO; n = 13; 4.9%), and the Region of the Americas (AMRO; n = 11; 4.2%). A Supplementary File contains all studies(with their reference list)included in this review according to WHO malaria-endemic regions (Table S1).

### 3.2. Global Plasmodium falciparum Drug Resistance Trends

**Figure 2:**
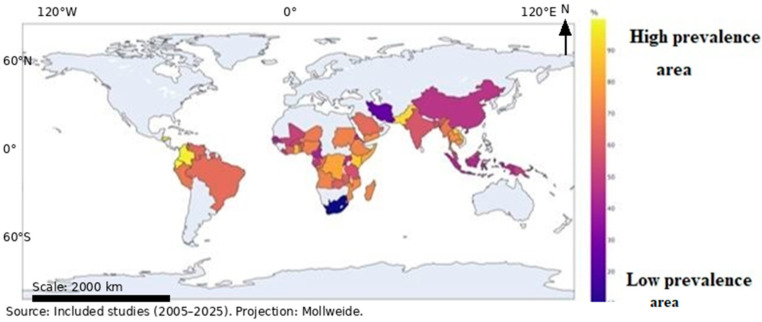
Global map for antimalarial drug resistance (the overall pooled prevalence).

The global distribution of resistance markers and prevalence data were summarized by country and WHO region. Geographic patterns were visualized using region- and country-level descriptive maps to illustrate the spatial distribution of resistance markers without inferential spatial modeling. Figure 2 illustrates the global distribution of malaria resistance markers. Areas with a high prevalence of resistance (70–90%) are indicated by yellow/light orange shading. These regions are considered hotspots where *P. falciparum* has been historically exposed to significant drug pressure.

This includes many countries in Central and West Africa, as well as some areas of South America (such as Brazil and Colombia). These high percentages suggest that first-line treatments are under severe threat in these locations because of widespread parasite resistance. Medium resistance levels (Orange/Reddish-Orange): These medium-to-high values (50–70%) are common across much of the sub-Saharan African malaria belt and parts of South-East Asia, indicating that drug resistance is an established and concerning factor in treatment policy. Lower resistance levels (Dark Blue/Purple): the lowest percentages (10–40%) are seen in certain countries in East Asia and Central Asia, suggesting that although the parasite is present, the prevalence of these specific resistance markers may be lower. Lastly, the unshaded regions, which cover North America, Europe, Australia, and Northern Asia, are not colored because they are generally not malaria-endemic regions. Nonetheless, malaria is imported to the mentioned regions, but there are no systematic studies that investigate resistance in these regions as done elsewhere. Consequently, in our review, we did not find any studies with primary data on *P. falciparum* drug resistance markers.

### 3.3. Pooled Prevalence of Antimalarial Drug Resistance Markers

Across all regions, the pooled prevalence of the five major *P. falciparum* drug resistance markers was high. The *pf*dhfr mutation exhibited the highest pooled prevalence (85.7%; 95% CI 80.2–89.9), followed by pfcrt (78.0%; 95% CI 72.0–83.0), pfdhps (73.7%; 95% CI 67.6–79.0), pfmdr1 (60.5%; 95% CI 54.7–66.0), and pfK13 (45.0%; 95% CI 36.2–54.1). Table 2 summarizes the pooled prevalence of each resistance marker. All markers showed very high heterogeneity (I^2^ > 98%), reflecting substantial between-study variability, likely driven by geographic, temporal, and methodological differences. All estimates were statistically significant (*p* < 0.001).

**Figure 3:**
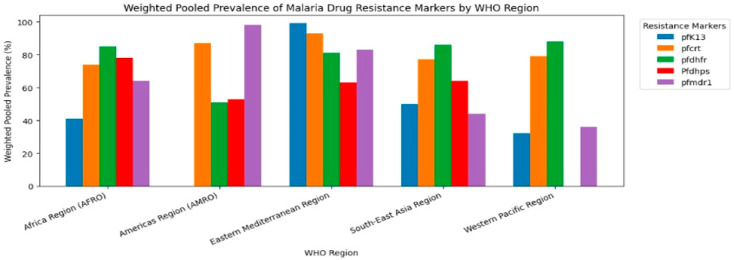
Pooled prevalence of resistance markers per WHO region.

When stratified by WHO region (Table 3) (Figure 3), pfmdr1 was most frequently reported in the African Region (k = 69), followed by pfcrt (k = 67) and *pf*dhfr (k = 44). In South-East Asia, *pf*crt (k = 25) and *pf*mdr1 (k = 24) dominated. The Americas reported limited but notable *pf*crt (k = 9) and *pf*mdr1 (k = 4) variants, whereas the Western Pacific Region reported fewer studies overall. Prevalence patterns varied significantly by WHO region. The pfcrt mutation rate was highest in the EMRO (93%), followed by the AMRO (87%), while remaining substantial in the AFRO (74%) and SEARO (77%). pfK13 mutations, markers of artemisinin resistance, were lower in the AFRO (40%) but nearly fixed in the EMRO (99%). The prevalence of *pf*dhfr mutations exceeded 80% in most regions, whereas pfmdr1 showed the greatest regional divergence, from 98% in the AMRO to 44% in the SEARO. Across all markers and regions, the between-study heterogeneity remained high (I^2^ > 95%).

### 3.4. Temporal Trends of P. falciparum Drug Resistance Markers (2005–2025)

Prespecified subgroup analyses were conducted by World Health Organization (WHO) region and study period. Temporal trends in resistance marker prevalence were explored using meta-regression, with the year of sample collection or publication year included as a continuous moderator when sufficient data were available.

**Figure 4:**
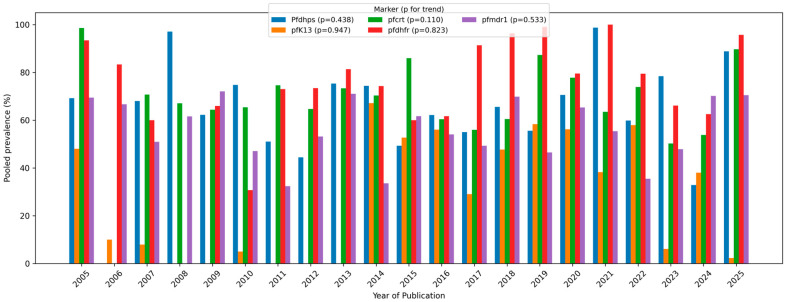
Temporal trends in pooled prevalence of five *P. falciparum* drug resistance markers between 2005 and 2025.

Temporal analysis (Figure 4) revealed a progressive increase in the pooled prevalence of most resistance markers over the past two decades. The *pf*dhfr and *pf*dhps mutation rates remained consistently high (82–43%), suggesting persistent selection pressure from the use of sulfadoxine–pyrimethamine. Conversely, *pf*crt prevalence declined modestly (52%) in some African subregions following chloroquine withdrawal but slightly increased after 2018, indicating possible re-emergence. The pfK13 mutations displayed an upward trajectory after 2012, corresponding to the scale-up of ACTs, particularly in South-East Asia and Africa.

### 3.5. Temporal Trends of P. falciparum Treatment Outcomes

**Figure 5:**
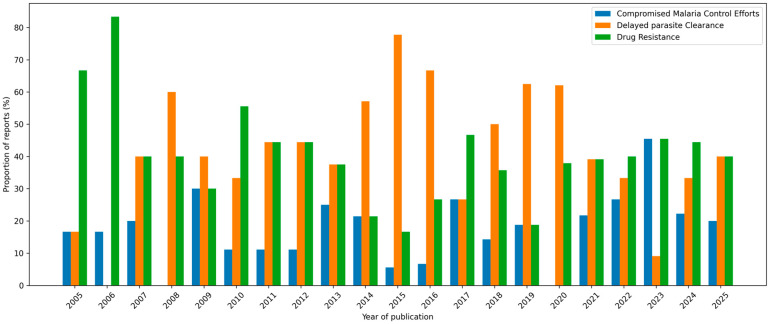
Temporal trends of *P. falciparum* treatment outcomes.

Analysis of the treatment outcomes between 2005 and 2025 revealed three major impacts: drug resistance, delayed parasite clearance, and compromised malaria control efforts (Figure 5). Treatment outcomes, including adequate clinical and parasitological response (ACPR) and delayed parasite clearance, were synthesized using a descriptive and narrative approach owing to substantial heterogeneity in outcome definitions, follow-up duration, and reporting methods across studies. Quantitative pooling of treatment outcomes was not performed unless the outcomes were defined and reported consistently across multiple studies.

Drug resistance remained consistently high (~40%) in cases of treatment failure. A prominent issue is the substantial variability but consistently high proportions (typically around 40%), suggesting an ongoing global concern. There were notable fluctuations in delayed parasite clearance, peaking around 2015 and 2019–2021. Conversely, compromised malaria control efforts displayed lower and more stable proportions (~30%), suggesting less research focus but ongoing relevance. The overall trend underscores the persistent global concern over resistance and its influence on treatment outcomes and malaria control, with research priorities shifting in response to new findings or local epidemics.

## 4. Discussion

Evidence from 261 studies across 64 countries was comprehensively synthesized in this systematic review and meta-analysis. Five genes, *pf*crt, pfmdr1, *pf*dhfr, *pf*dhps, and *pf*k13, emerged as key molecular markers of antimalarial drug resistance. The pooled prevalence ranged from 44.9% for pfk13 to 85.7% for *pf*dhfr, underscoring the persistence and global spread of *P. falciparum* drug resistance in the region. These results confirm that antimalarial resistance remains an evolving global health threat shaped by both historical drug use and ongoing selective pressure from current therapies.

Distinct regional differences were observed in the distribution and prevalence of the resistance markers. In the African region, pfmdr1 (69 studies) and *pf*crt (67) were most frequently reported, with prevalence ranging from 40.5% for pfk13 to 84.9% for pfdhfr. The Americas recorded very high levels of *pf*crt (87.5%) and pfmdr1 (97.9%), whereas the South-East Asia Region exhibited the highest prevalence of *pf*crt (77.4%) and pfk13 (49.9%). Pfcrt was also predominant in the EMRO and WPRO. These findings are consistent with WHO regional reports indicating that *pf*crt haplotypes persist in South America and that pfdhfr/pfdhps mutations remain widespread in Africa despite chloroquine withdrawal. This persistence shows that drug resistance markers may remain stable within parasite populations even after drug withdrawal.

According to Awasthi et al. (2023) [20], resistant haplotypes may persist even in the absence of direct drug pressure, as seen in the sustained *pf*crt prevalence (≈78%) in West and Central Africa despite chloroquine withdrawal. The moderate yet clinically significant pfk13 prevalence (44.9%) emphasizes the gradual but alarming emergence of artemisinin resistance, whereas pfmdr1 (60.5%) emphasizes its function in modulating ACT partner-drug efficacy. Thus, the coexistence of legacy resistance (to CQ and SP) and emerging resistance (to ACTs) poses a dual challenge to malaria control. This implies that local antimalarial drug-use histories strongly influence regional resistance patterns, reinforcing the need for context-specific treatment policies rather than uniform global ones [21,22].

Although *pf*dhfr, *pf*crt, and *pf*dhps consistently exhibited high pooled prevalence across regions, substantial between-study variability persisted, reflecting differences in drug-pressure histories, sampling frames, and laboratory methods. Although pfdhfr, pfcrt, and pfdhps consistently exhibited the highest pooled prevalence across all WHO regions compared to the other markers, all markers demonstrated very high heterogeneity (I^2^ > 95%), indicating that strong geographic, temporal, and methodological variation, and differences in study design, molecular methods, sampling frames (e.g., clinical vs. asymptomatic community infections), and time lags also drive much of the heterogeneity among studies. In addition, the very high heterogeneity (I^2^ > 95%) indicates substantial variation in resistance marker prevalence across settings; thus, pooled estimates should be interpreted as summary averages rather than universally generalizable point estimates. Therefore, we used random-effects models, which assume that true effects vary between studies, and we emphasized regional and temporal subgroup patterns as more actionable for policy-making.

The continued selective pressure from sulfadoxine–pyrimethamine (SP), which is still widely used in Africa for intermittent preventive treatment in pregnancy (IPTp) and seasonal malaria chemoprevention in Africa, explains the dominance of pfdhfr and pfdhps [23,24]. Similarly, some studies have documented persistently high frequencies of pfdhfr/pfdhps mutations, especially in East Africa, which are strongly correlated with treatment failure for SP [25,26].

Temporal trends reveal complex evolutionary dynamics. pfk13 prevalence rose substantially after 2010, peaking around 2019, corresponding to widespread ACT use in the SEAROs [27,28]. The post-2012 rise in pfk13 underscores the risk of broader expansion of artemisinin partial resistance into new regions, while patterns suggesting the re-emergence of older resistance markers highlight the need for sustained surveillance even after policy shifts. Future work should prioritize standardized longitudinal genomic surveillance linked to therapeutic efficacy outcomes to anticipate and mitigate emerging scenarios of treatment failure. The decline of pfk13 between 2023 and 2025 may suggest reduced selective pressure or fitness costs outside Asia. pfcrt declined after chloroquine withdrawal but resurged post-2018, likely driven by amodiaquine cross-resistance or residual genetic selection [29]. pfdhps and pfmdr1 exhibited cyclical fluctuations, each showing temporal declines followed by rebounds. A high prevalence of dhfr/dhps mutations raises concerns about the effectiveness of SP-based preventive strategies (e.g., IPTp) in some settings, while increasing pfk13 prevalence signals heightened risk of delayed parasite clearance and potential ACT efficacy erosion. These findings support the alignment of molecular surveillance with therapeutic efficacy studies and the updating of treatment/prevention policies where resistance thresholds are exceeded. These patterns demonstrate how *P. falciparum* evolution continues to follow past and present drug pressures, highlighting the ongoing risk of ACT resistance and underscoring the need for continued molecular surveillance and a more diverse approach to drug policies. Collectively, these heterogeneous patterns demonstrate that *P. falciparum* continues to evolve in response to past and current antimalarial interventions. This underscores the importance of continuous molecular surveillance, ongoing review of treatment guidelines, and research and development of new therapeutic strategies to prevent widespread ACT failure.

Most included studies were observational and cross-sectional, which limits causal inference and reduces consistency in linking resistance markers to treatment outcomes. In addition, treatment outcome measures were synthesized narratively because definitions, follow-up duration, and reporting formats varied across studies, reducing comparability. Differences in laboratory genotyping approaches and marker definitions across studies may have introduced measurement variability and misclassification. Finally, residual confounding (e.g., transmission intensity, prior drug exposure, and concurrent interventions) could not be fully accounted for, which may influence observed marker prevalence patterns and trends.

## 5. Conclusions

This review highlighted the global distribution and temporal trends and prevalence of drug resistance markers in *P. falciparum* over two decades. Pfdhfr and pfdhps mutations persist and maintain resistance to sulfadoxine–pyrimethamine, whereas pfcrt remains active even after withdrawal of chloroquine use. The heterogeneous distribution and rise in pfk13 mutations signal a concerning spread of artemisinin resistance. The pfmdr1 variation reflects local drug-use histories and its function in partner-drug tolerance. These results demonstrate that antimalarial drug resistance remains a dynamic and widespread challenge that is significantly influenced by geographical location. To cope with these challenges, regulatory authorities must maintain efforts to control and eradicate malaria, regular molecular surveillance must be incorporated into national control programs, treatment recommendations should take into account local resistance profiles, and funding for innovative therapeutic approaches and adaptive techniques is crucial. Moreover, molecular surveillance data provide a clear understanding of resistance evolution and thus must be used to inform malaria treatment policies; thus, periodic molecular marker surveys should be aligned with national therapeutic efficacy monitoring. Countries should integrate standardized panels for resistance markers or emerging methods like cost-effective long-read sequencing, such as Oxford Nanopore (ONT), and molecular data should be open for sharing and linked to clinical outcomes. Countries should also pay attention to early indications of ACT partner-drug resistance. Novel drug combinations should be investigated, and treatment protocols should be modified before resistance solidifies.

## Figures and Tables

**Table 1:** Searching strategies for databases.

S/N	Database	Searching String	Number of First Retrievals	Searching Date
1	PubMed	(“*Plasmodium falciparum*”[tiab] OR “*falciparum* malaria”[tiab]) AND (“drug resistance”[tiab] OR “antimalarial resistance”[tiab] OR resistance[tiab] OR mutation[tiab] OR polymorphism[tiab]) AND (“pfcrt”[tiab] OR “pfmdr1”[tiab] OR “pfdhfr”[tiab] OR “pfdhps”[tiab] OR “kelch13”[tiab] OR “k13”[tiab]) AND (prevalence[tiab] OR distribution[tiab] OR epidemiology[tiab] OR incidence[tiab] OR “treatment outcome”[tiab] OR efficacy[tiab] OR “drug response”[tiab]) AND (“artemisinin”[tiab] OR “chloroquine”[tiab] OR “sulfadoxine-pyrimethamine”[tiab] OR ACT[tiab] OR amodiaquine[tiab] OR mefloquine[tiab] OR lumefantrine[tiab] OR quinine[tiab]) AND (“Clinical Trial”[Publication Type] OR “Observational Study”[Publication Type] OR “Clinical Study”[Publication Type] OR survey[tiab] OR “field study”[tiab])	93	25 August 2025
2	Google scholar	(“*Plasmodium falciparum*” OR “*falciparum* malaria”) AND (“drug resistance” OR “antimalarial resistance”) AND (“pfcrt” OR “pfmdr1” OR “pfdhfr” OR “pfdhps” OR “kelch13” OR “k13”) AND (mutation OR polymorphism) AND (prevalence OR distribution OR epidemiology OR incidence OR “treatment outcome” OR efficacy OR “drug response”) AND (“clinical study” OR “observational study” OR “field study” OR trial OR survey) AND (“artemisinin” OR “chloroquine” OR “sulfadoxine-pyrimethamine” OR ACT)	3520 200 current ones were exported from Publish or Perish	27 August 2025
3	WEB of Sciences	(“*Plasmodium falciparum*” OR “*falciparum* malaria”) (Topic) AND (“drug resistance” OR “antimalarial resistance”) (Topic) AND (“pfcrt” OR “pfmdr1” OR “pfdhfr” OR “pfdhps” OR “kelch13” OR “k13”) (Topic) AND (mutation OR polymorphism) (Topic) AND (prevalence OR “treatment outcome” OR efficacy) (Topic) AND (“artemisinin” OR “chloroquine” OR “sulfadoxine-pyrimethamine” OR amodiaquine OR mefloquine OR lumefantrine OR quinine) (Topic)	1520	27 August 2025
4	Scopus	(“*Plasmodium falciparum*” OR “*falciparum* malaria”) AND TITLE-ABS-KEY (“drug resistance” OR “antimalarial resistance”) AND TITLE-ABS-KEY (“pfcrt” OR “pfmdr1” OR “pfdhfr” OR “pfdhps” OR “kelch13” OR “k13”) AND TITLE-ABS-KEY (mutation OR polymorphism) AND TITLE-ABS-KEY (prevalence OR “treatment outcome” OR efficacy) AND TITLE-ABS-KEY (“artemisinin” OR “chloroquine” OR “sulfadoxine-pyrimethamine” OR amodiaquine OR mefloquine OR lumefantrine OR quinine)	65	27 August 2025

**Table 2:** Global pooled prevalence of drug resistance markers.

Resistance Marker	K (Studies)	Pooled Prevalence (%)	95% CI	Tau^2^	I^2^ (%)	Cochran’s Q (df)	*p*-Value
*pf*dhps	60	73.7	67.6–79.0	1.269742	98.7	4407.08 (59)	<0.001
*pf*K13	66	45	36.2–54.1	2.123132	99.3	9708.85 (65)	<0.001
*pf*crt	115	78	72.0–83.0	2.782854	99.2	13,653.54 (114)	<0.001
*pf*dhfr	66	85.7	80.2–89.9	2.333511	99.6	15,644.70 (65)	<0.001
*pf*mdr1	110	60.5	54.7–66.0	1.459759	99.1	12,518.59 (109)	<0.001

**Key: K:** Number of studies examined for each marker among total (N); **CI:** Confidence interval. for each resistance marker; **I^2^**: the percentage of the variability due to heterogeneity. Note: Pooled prevalence estimates were obtained using random-effects meta-analysis. Between-study heterogeneity was assessed using Cochran’s Q and quantified using the I^2^ statistic, with between-study variance estimated as τ^2^. All Q tests indicated statistically significant heterogeneity (*p* < 0.001). In the Supplementary Materials, we have included a funnel plot with additional information for standard error estimates (study-level standard errors and weights are shown in the forest plots).

**Table 3:** Regional pooled prevalence of drug resistance markers.

Resistance Marker	Region	K	Pooled Prevalence	Cl	Cl	I^2^_%
*pfdhps*	AFRO	42	78%	70	83	99
	AMRO	2	53%	12	90	96
	EMRO	5	63%	47	76	96
	SEARO	11	64%	45	79	98
*pfk13*	AFRO	43	40%	29	53	99
	EMRO	2	99%	97	99	0
	SEARO	18	50%	34	65	99
	EPRO	3	27%	87	57	96
*pfcrt*	AFRO	67	74%	64	81	99
	AMRO	9	87%	70	95	93
	EMRO	6	93%	90	98	96
	SEARO	25	77%	65	86	98
	WPRO	8	79%	68	87	97
*pfdhfr*	AFRO	44	85%	78	89	99
	AMRO	2	51%	35	66	66
	EMRO	6	81%	62	91	94
	SEARO	13	86%	78	91	97
	WPRO	1	88%	80	93	0
*pfmdr1*	AFRO	69	63%	55	69	99
	AMRO	4	98%	79	99	83
	EMRO	8	83%	63	92	96
	SEARO	24	44%	35	52	98
	WPRO	5	47%	17	79	99.5

Key: AFRO = African Region; AMRO = Region of the Americas; EMRO = Eastern Mediterranean Region; SEARO = South-East Asia Region; WPRO = Western Pacific Region.

## Data Availability

The data used in this review are available and are submitted in the Supplementary Files.

## Supplementary Materials

The following supporting information can be downloaded at: https://www.mdpi.com/article/10.3390/pathogens15020179/s1, File S1: PRISMA 2020 Checklist; File S2: File of Excluded studies; File S3: File of final included studies; File S4: Table of Quality Assessment/Risk of Bias assessment; Table S1: Distribution of Included Studies, Resistance Markers and Antimalarial Drugs by WHO Regions; Figure S1: Funnel plots for global prevalence of *Plasmodium falciparum* resistance markers.

## Abbreviations

The following abbreviations are used in this manuscript:

CRT	Chloroquine resistance transporter
DHPS	Dihydropteroate synthase
MDR1	Multidrug resistance 1
DHFR	Dihydrofolate reductase
SP	Sulfadoxine–pyrimethamine
ACT	Artemisinin-based combination therapy
CQ	Chloroquine
