# Supplementary material for: The Global Landscape of Plasmodium falciparum Drug Resistance Markers, 2005–2025: A Systematic Review and Meta-Analysis
Source: Pathogens. 2026 Feb 6;15(2):179. doi: 10.3390/pathogens15020179 (PMC12943415; doi:10.3390/pathogens15020179)

Supplementary Figure S1. Funnel plots for global prevalence of Plasmodium falciparum resistance markers.

Each panel shows the pooled effect (solid vertical line) and 95% pseudo-confidence limits (dashed lines).

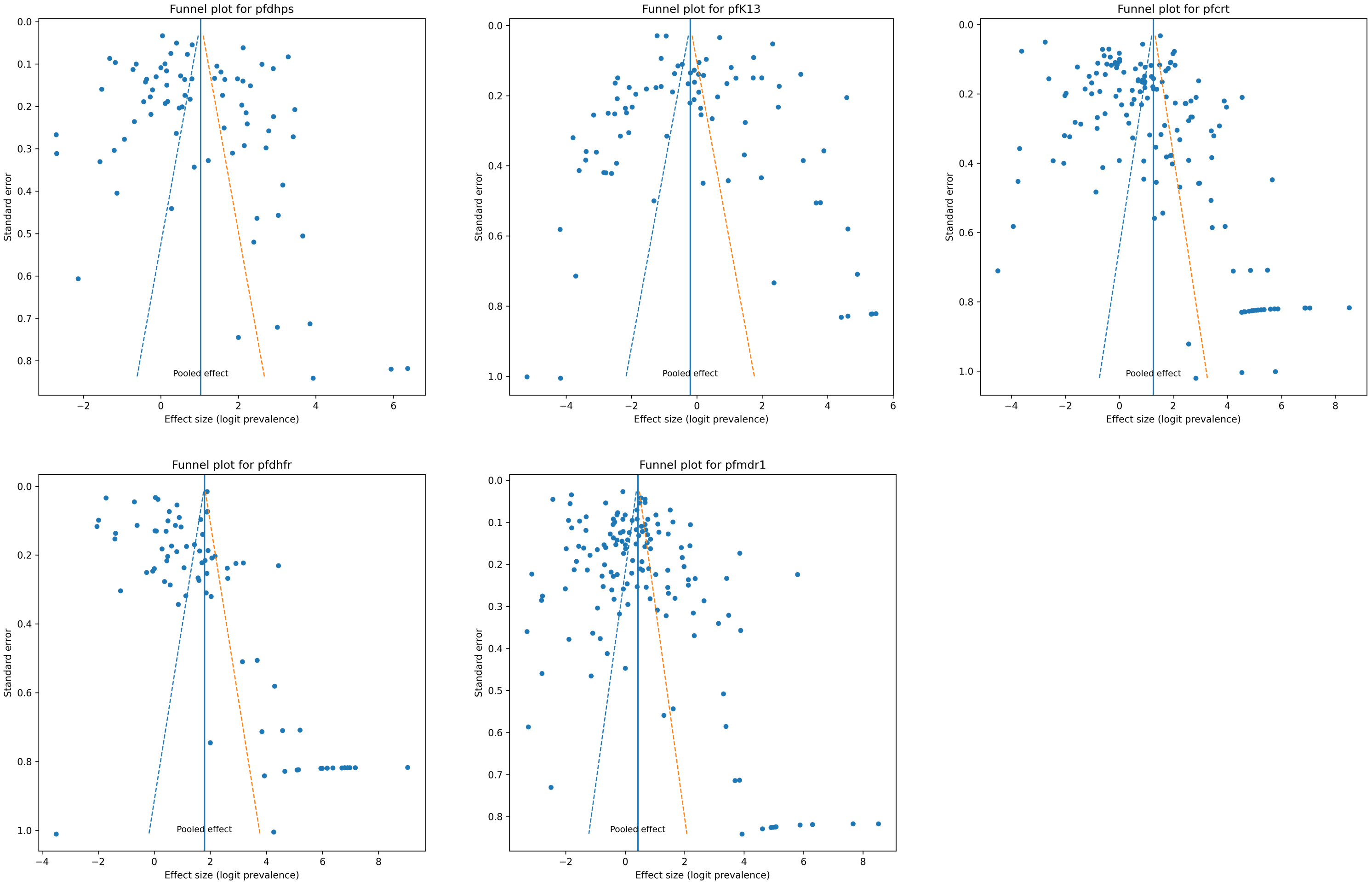

Supplement: Supplementary file 1 [file pathogens-15-00179-s001.zip › Figure S1.pdf]
