# Supplementary material for: The Global Landscape of Plasmodium falciparum Drug Resistance Markers, 2005–2025: A Systematic Review and Meta-Analysis
Source: Pathogens. 2026 Feb 6;15(2):179. doi: 10.3390/pathogens15020179 (PMC12943415; doi:10.3390/pathogens15020179)
Supplement: Supplementary file 1 [file pathogens-15-00179-s001.zip › Table S1.pdf]

Table S1: Distribution of Included Studies, Resistance Markers and Antimalarial Drugs by WHO Regions

**Region**	**Countries sampled**	**Number of Studies Involved (%)**	**Key resistance markers**	**References**
Africa Region	Angola, Benin, Burkina Faso, Cameroon, Cape Verde, Central Africa, Comoros, Cote d'Ivoire, Democratic Republic of Congo, Equatorial Guinea, Eritrea, Ethiopia, Gabon, Ghana, Guinea-Bissau, Kenya, Liberia, Madagascar, Malawi, Mali, Mozambique, Guinea, Niger, Nigeria, Congo, Rwanda, Sao Tome et Principe, Senegal, Somalia, South Africa, Swaziland, Tanzania, Togo, Uganda, Zambia	166 (63.6%)	*pfcrt, pfmdr1 pfdhfr, pfk13, and pfdhps*	[1–166]
Region of the Americas	Brazil, Colombia, Ecuador, French Guinea, Honduras, Peru, Suriname, Venezuela	11 (4.2%)	*pfcrt, pfmdr1 pfdhfr, pfk13, and pfdhps*	[167–177]
Eastern Mediterranean Region (EMRO)	Iran, Pakistan, Saudi Arabia Somalia, Sudan, Yemen	13 (4.3%)	*pfcrt, pfmdr1 pfdhfr, pfk13, and pfdhps*	[178–190]
South-East Asia Region	Cambodia, China, Colombia, Timor-Leste, India, Indonesia, Myanmar, Thailand	55 (21%)	*pfcrt, pfmdr1 pfdhfr, pfk13, and pfdhps*	[191–245]
Western Pacific Region	Laos, Malaysia, Papua New Guinea, Solomon Islands, Viet Nam	16 (6.1%)	*pfcrt, pfmdr1 pfdhfr, pfk13, and pfdhps*	[246–261]

REFERENCES

1. Fançony C, Fortes-Gabriel E, Zage F, ... Artemether-Lumefantrine Treatment Selects Plasmodium falciparum Multidrug Resistance 1 (pfmdr1) Increased Copy Number Among African Malaria Infections. … Journal of Infectious … [Internet] 2025;(Query date: 2025-07-02 12:44:41). Available from: https://academic.oup.com/jid/advance-article-abstract/doi/10.1093/infdis/jiaf155/8095636

2. Gendrot M, Delandre O, Robert MG, Foguim FT, Benoit N, Amalvict R, et al. Absence of Association between Methylene Blue Reduced Susceptibility and Polymorphisms in 12 Genes Involved in Antimalarial Drug Resistance in African Plasmodium falciparum. Pharmaceuticals (Basel) 2021;14(4).

3. Gharbi M, Flegg JA, Hubert V, Kendjo E, Metcalf JE, Bertaux L, et al. Longitudinal study assessing the return of chloroquine susceptibility of Plasmodium falciparum in isolates from travellers returning from West and Central Africa, 2000-2011. Malar J 2013;12:35.

4. Ursing J, Rombo L, Bergqvist Y, Rodrigues A, Kofoed PE. High-Dose Chloroquine for Treatment of Chloroquine-Resistant Plasmodium falciparum Malaria. J Infect Dis 2016;213(8):1315–21.

5. Duah NO, Matrevi SA, De Souza DK, Binnah DD, Tamakloe MM, Opoku VS, et al. Increased pfmdr1 gene copy number and the decline in pfcrt and pfmdr1 resistance alleles in Ghanaian Plasmodium falciparum isolates after the change of anti-malarial drug treatment policy. Malar J 2013;12(1):377.

6. Gupta H, Macete E, Bulo H, Salvador C, Warsame M, Carvalho E, et al. Drug-Resistant Polymorphisms and Copy Numbers in Plasmodium falciparum, Mozambique, 2015. Emerg Infect Dis 2018;24(1):40–8.

7. Niba PTN, Nji AM, Chedjou JPK, Hansson H, Hocke EF, Ali IM, et al. Evolution of Plasmodium falciparum antimalarial drug resistance markers post-adoption of artemisinin-based combination therapies in Yaounde, Cameroon. Int J Infect Dis 2023;132:108–17.

8. Otienoburu SD, Maïga-Ascofaré O, Schramm B, Jullien V, Jones JJ, Zolia YM, et al. Selection of Plasmodium falciparum pfcrt and pfmdr1 polymorphisms after treatment with artesunate-amodiaquine fixed dose combination or artemether-lumefantrine in Liberia. Malar J 2016;15(1):452.

9. Koko VS, Warsame M, Vonhm B, Jeuronlon MK, Menard D, Ma L, et al. Artesunate-amodiaquine and artemether-lumefantrine for the treatment of uncomplicated falciparum malaria in Liberia: in vivo efficacy and frequency of molecular markers. Malar J 2022;21(1):134.

10. Koukouikila-Koussounda F, Bakoua D, Fesser A, Nkombo M, Vouvoungui C, Ntoumi F. High prevalence of sulphadoxine-pyrimethamine resistance-associated mutations in Plasmodium falciparum field isolates from pregnant women in Brazzaville, Republic of Congo. Infect Genet Evol 2015;33:32–6.

11. Koumba Lengongo JV, Ndiaye YD, Tshibola Mbuyi ML, Ndong Ngomo JM, Ndiaye D, Bouyou Akotet MK, et al. Increased Frequency of Pfdhps A581G Mutation in Plasmodium falciparum Isolates from Gabonese HIV-Infected Individuals. Malar Res Treat 2019;2019:9523259.

12. L'Episcopia M, Kelley J, Patel D, Schmedes S, Ravishankar S, Menegon M, et al. Targeted deep amplicon sequencing of kelch 13 and cytochrome b in Plasmodium falciparum isolates from an endemic African country using the Malaria Resistance Surveillance (MaRS) protocol. Parasit Vectors 2020;13(1):137.

13. Li J, Chen J, Xie D, Monte-Nguba S, ... High prevalence of pfmdr1 N86Y and Y184F mutations in Plasmodium falciparum isolates from Bioko island, Equatorial Guinea. Pathogens and global … [Internet] 2014;(Query date: 2025-07-02 12:44:41). Available from: https://www.tandfonline.com/doi/abs/10.1179/2047773214Y.0000000158

14. Lin LY, Li J, Huang HY, Liang XY, Jiang TT, Chen JT, et al. Trends in Molecular Markers Associated with Resistance to Sulfadoxine-Pyrimethamine (SP) Among Plasmodium falciparum Isolates on Bioko Island, Equatorial Guinea: 2011-2017. Infect Drug Resist 2020;13:1203–12.

15. Lo AC, Faye B, Ba EH, Cisse B, Tine R, Abiola A, et al. Prevalence of molecular markers of drug resistance in an area of seasonal malaria chemoprevention in children in Senegal. Malar J 2013;12:137.

16. Lobo E, de Sousa B, Rosa S, Figueiredo P, Lobo L, Pateira S, et al. Prevalence of pfmdr1 alleles associated with artemether-lumefantrine tolerance/resistance in Maputo before and after the implementation of artemisinin-based combination therapy. Malar J 2014;13:300.

17. Loon W van, Oliveira R, Bergmann C, ... In vitro confirmation of artemisinin resistance in Plasmodium falciparum from patient isolates, Southern Rwanda, 2019. Emerging infectious … [Internet] 2022;(Query date: 2025-07-02 12:44:41). Available from: https://pmc.ncbi.nlm.nih.gov/articles/PMC8962885/

18. Malmberg M, Ferreira PE, Tarning J, Ursing J, Ngasala B, Björkman A, et al. Plasmodium falciparum drug resistance phenotype as assessed by patient antimalarial drug levels and its association with pfmdr1 polymorphisms. J Infect Dis 2013;207(5):842–7.

19. Efficacy of Artemether-Lumefantrine and Dihydroartemisinin-Piperaquine for the Treatment of Uncomplicated Plasmodium falciparum Malaria among Children in Western Kenya, 2016 to 2017 - PubMed [Internet]. [cited 2025 Sep 26];Available from: https://pubmed.ncbi.nlm.nih.gov/36036611/

20. Nsobya SL, Dokomajilar C, Joloba M, Dorsey G, Rosenthal PJ. Resistance-mediating Plasmodium falciparum pfcrt and pfmdr1 alleles after treatment with artesunate-amodiaquine in Uganda. Antimicrob Agents Chemother 2007;51(8):3023–5.

21. Tacoli C, Gai PP, Bayingana C, Sifft K, Geus D, Ndoli J, et al. Artemisinin Resistance-Associated K13 Polymorphisms of Plasmodium falciparum in Southern Rwanda, 2010-2015. Am J Trop Med Hyg 2016;95(5):1090–3.

22. Tahar R, Basco LK. Molecular epidemiology of malaria in Cameroon. XXVII. Clinical and parasitological response to sulfadoxine-pyrimethamine treatment and Plasmodium falciparum dihydrofolate reductase and dihydropteroate synthase alleles in Cameroonian children. Acta Trop 2007;103(2):81–9.

23. Tumwebaze P, Tukwasibwe S, Taylor A, Conrad M, Ruhamyankaka E, Asua V, et al. Changing Antimalarial Drug Resistance Patterns Identified by Surveillance at Three Sites in Uganda. J Infect Dis 2017;215(4):631–5.

24. M van L, R van der M, M L, A O, E B, C B, et al. Markers of sulfadoxine-pyrimethamine resistance in Eastern Democratic Republic of Congo; implications for malaria chemoprevention. Malaria journal [Internet] 2019 [cited 2025 Sep 26];18(1). Available from: https://pubmed.ncbi.nlm.nih.gov/31852480/

25. van Loon W, Schallenberg E, Igiraneza C, Habarugira F, Mbarushimana D, Nshimiyimana F, et al. Escalating Plasmodium falciparum K13 marker prevalence indicative of artemisinin resistance in southern Rwanda. Antimicrob Agents Chemother 2024;68(1):e0129923.

26. van Loon W, Schallenberg E, Mande E, Musinguzi P, Ngobi P, Atukunda S, et al. Plasmodium falciparum Kelch-13 artemisinin partial resistance markers in Fort Portal, Western Uganda, 2024. Antimicrob Agents Chemother 2025;69(6):e0175524.

27. Asua V, Vinden J, Conrad MD, Legac J, Kigozi SP, Kamya MR, et al. Changing Molecular Markers of Antimalarial Drug Sensitivity across Uganda. Antimicrob Agents Chemother 2019;63(3).

28. Baraka V, Tinto H, Valea I, Fitzhenry R, ... In Vivo Selection of Plasmodium falciparum Pfcrt and Pfmdr1 Variants by Artemether-Lumefantrine and Dihydroartemisinin-Piperaquine in Burkina Faso. Antimicrobial agents … [Internet] 2015;(Query date: 2025-07-02 12:44:41). Available from: https://journals.asm.org/doi/abs/10.1128/aac.03647-14

29. Voumbo-Matoumona DF, Kouna LC, Madamet M, Maghendji-Nzondo S, Pradines B, Lekana-Douki JB. Prevalence of Plasmodium falciparum antimalarial drug resistance genes in Southeastern Gabon from 2011 to 2014. Infect Drug Resist 2018;11:1329–38.

30. Wang X, Zhang X, Chen H, Lu Q, Ruan W, Chen Z. Molecular Determinants of Sulfadoxine-Pyrimethamine Resistance in Plasmodium falciparum Isolates from Central Africa between 2016 and 2021: Wide Geographic Spread of Highly Mutated Pfdhfr and Pfdhps Alleles. Microbiol Spectr 2022;10(5):e0200522.

31. Wurtz N, Fall B, Pascual A, Diawara S, Sow K, Baret E, et al. Prevalence of molecular markers of Plasmodium falciparum drug resistance in Dakar, Senegal. Malar J 2012;11:197.

32. Xu C, Wei Q, Yin K, Sun H, Li J, Xiao T, et al. Surveillance of Antimalarial Resistance Pfcrt, Pfmdr1, and Pfkelch13 Polymorphisms in African Plasmodium falciparum imported to Shandong Province, China. Sci Rep 2018;8(1):12951.

33. Kiwuwa MS, Byarugaba J, Wahlgren M, Kironde F. Detection of copy number variation and single nucleotide polymorphisms in genes involved in drug resistance and other phenotypic traits in P. falciparum clinical isolates collected from Uganda. Acta Trop 2013;125(3):269–75.

34. Kiaco K, Teixeira J, Machado M, do Rosário V, Lopes D. Evaluation of artemether-lumefantrine efficacy in the treatment of uncomplicated malaria and its association with pfmdr1, pfatpase6 and K13-propeller polymorphisms in Luanda, Angola. Malar J 2015;14:504.

35. Diakité SAS, Traoré K, Sanogo I, Clark TG, Campino S, Sangaré M, et al. A comprehensive analysis of drug resistance molecular markers and Plasmodium falciparum genetic diversity in two malaria endemic sites in Mali. Malar J 2019;18(1):361.

36. Kateera F, Nsobya SL, Tukwasibwe S, Hakizimana E, Mutesa L, Mens PF, et al. Molecular surveillance of Plasmodium falciparum drug resistance markers reveals partial recovery of chloroquine susceptibility but sustained sulfadoxine-pyrimethamine resistance at two sites of different malaria transmission intensities in Rwanda. Acta Trop 2016;164:329–36.

37. Schunk M, Kumma W, Miranda I, Osman M, ... High prevalence of drug-resistance mutations in Plasmodium falciparum and Plasmodium vivax in southern Ethiopia. Malaria Journal [Internet] 2006;(Query date: 2025-07-02 12:44:41). Available from: https://link.springer.com/article/10.1186/1475-2875-5-54

38. Apinjoh TO, Mugri RN, Miotto O, Chi HF, Tata RB, Anchang-Kimbi JK, et al. Molecular markers for artemisinin and partner drug resistance in natural Plasmodium falciparum populations following increased insecticide treated net coverage along the slope of mount Cameroon: cross-sectional study. Infect Dis Poverty 2017;6(1):136.

39. Ojurongbe O, Ogungbamigbe TO, Fagbenro-Beyioku AF, Fendel R, Kremsner PG, Kun JFJ. Rapid detection of Pfcrt and Pfmdr1 mutations in Plasmodium falciparum isolates by FRET and in vivo response to chloroquine among children from Osogbo, Nigeria. Malar J 2007;6:41.

40. Mensah BA, Aydemir O, Myers-Hansen JL, Opoku M, Hathaway NJ, Marsh PW, et al. Antimalarial Drug Resistance Profiling of Plasmodium falciparum Infections in Ghana Using Molecular Inversion Probes and Next-Generation Sequencing. Antimicrob Agents Chemother 2020;64(4).

41. Pacheco MA, Schneider KA, Cheng Q, Munde EO, Ndege C, Onyango C, et al. Changes in the frequencies of Plasmodium falciparum dhps and dhfr drug-resistant mutations in children from Western Kenya from 2005 to 2018: the rise of Pfdhps S436H. Malar J 2020;19(1):378.

42. Ishengoma DS, Mandara CI, Francis F, Talundzic E, Lucchi NW, Ngasala B, et al. Efficacy and safety of artemether-lumefantrine for the treatment of uncomplicated malaria and prevalence of Pfk13 and Pfmdr1 polymorphisms after a decade of using artemisinin-based combination therapy in mainland Tanzania. Malar J 2019;18(1):88.

43. Tola M, Ajibola O, Idowu ET, Omidiji O, Awolola ST, Amambua-Ngwa A. Molecular detection of drug resistant polymorphisms in Plasmodium falciparum isolates from Southwest, Nigeria. BMC Res Notes 2020;13(1):497.

44. Bonizzoni M, Afrane Y, Baliraine F, ... Genetic structure of Plasmodium falciparum populations between lowland and highland sites and antimalarial drug resistance in Western Kenya. Infection, Genetics and … [Internet] 2009;(Query date: 2025-07-02 12:44:41). Available from: https://www.sciencedirect.com/science/article/pii/S1567134809000860

45. Osborne A, Phelan JE, Kaneko A, Kagaya W, Chan C, Ngara M, et al. Drug resistance profiling of asymptomatic and low-density Plasmodium falciparum malaria infections on Ngodhe island, Kenya, using custom dual-indexing next-generation sequencing. Sci Rep 2023;13(1):11416.

46. Prevalence of Plasmodium falciparum parasites harbouring chloroquine-resistant but not artemisinin-resistant alleles in Busia County, Western Kenya | Malaria Journal | Full Text [Internet]. [cited 2025 Sep 26];Available from: https://malariajournal.biomedcentral.com/articles/10.1186/s12936-025-05486-4

47. Abera D, Kibet CK, Degefa T, Amenga-Etego L, Bargul JL, Golassa L. Genomic analysis reveals independent evolution of Plasmodium falciparum populations in Ethiopia. Malar J 2021;20(1):129.

48. Lucchi NW, Komino F, Okoth SA, Goldman I, Onyona P, Wiegand RE, et al. In Vitro and Molecular Surveillance for Antimalarial Drug Resistance in Plasmodium falciparum Parasites in Western Kenya Reveals Sustained Artemisinin Sensitivity and Increased Chloroquine Sensitivity. Antimicrob Agents Chemother 2015;59(12):7540–7.

49. Alifrangis M, Lusingu JP, Mmbando B, Dalgaard MB, Vestergaard LS, Ishengoma D, et al. Five-year surveillance of molecular markers of Plasmodium falciparum antimalarial drug resistance in Korogwe District, Tanzania: accumulation of the 581G mutation in the P. falciparum dihydropteroate synthase gene. Am J Trop Med Hyg 2009;80(4):523–7.

50. Chen YA, Shiu TJ, Tseng LF, Cheng CF, Shih WL, de Assunção Carvalho AV, et al. Dynamic changes in genetic diversity, drug resistance mutations, and treatment outcomes of falciparum malaria from the low-transmission to the pre-elimination phase on the islands of São Tomé and Príncipe. Malar J 2021;20(1):467.

51. Uwimana A, Umulisa N, Venkatesan M, Svigel SS, Zhou Z, Munyaneza T, et al. Association of *Plasmodium falciparum kelch13* R561H genotypes with delayed parasite clearance in Rwanda: an open-label, single-arm, multicentre, therapeutic efficacy study.

The Lancet Infectious Diseases [Internet] 2021 [cited 2025 Jul 22];21(8):1120–8. Available from: https://www.sciencedirect.com/science/article/pii/S1473309921001420

52. Maiga H, Grivoyannis A, Sagara I, Traore K, Traore OB, Tolo Y, et al. Selection of pfcrt K76 and pfmdr1 N86 Coding Alleles after Uncomplicated Malaria Treatment by Artemether-Lumefantrine in Mali. Int J Mol Sci 2021;22(11).

53. Fukuda N, Tachibana SI, Ikeda M, Sakurai-Yatsushiro M, Balikagala B, Katuro OT, et al. Ex vivo susceptibility of Plasmodium falciparum to antimalarial drugs in Northern Uganda. Parasitol Int 2021;81:102277.

54. Cuu G, Asua V, Tukwasibwe S, Nsobya SL, Nanteza A, Kimuda MP, et al. Associations between Aminoquinoline Resistance Genotypes and Clinical Presentations of Plasmodium falciparum Infection in Uganda. Antimicrob Agents Chemother 2020;64(10):e00721-20.

55. Ross LS, Dhingra SK, Mok S, Yeo T, Wicht KJ, Kümpornsin K, et al. Emerging Southeast Asian PfCRT mutations confer Plasmodium falciparum resistance to the first-line antimalarial piperaquine. Nat Commun 2018;9(1):3314.

56. Asare KK, Boampong JN, Duah NO, Afoakwah R, Sehgal R, Quashie NB. Synergism between Pfcrt and Pfmdr1 genes could account for the slow recovery of chloroquine sensitive Plasmodium falciparum strains in Ghana after chloroquine withdrawal. J Infect Public Health 2017;10(1):110–9.

57. Tumwebaze P, Conrad M, Walakira A, ... Impact of antimalarial treatment and chemoprevention on the drug sensitivity of malaria parasites isolated from Ugandan children. Antimicrobial agents … [Internet] 2015;(Query date: 2025-07-02 12:44:41). Available from: https://journals.asm.org/doi/abs/10.1128/aac.05141-14

58. Cheruiyot J, Ingasia LA, Omondi AA, Juma DW, Opot BH, Ndegwa JM, et al. Polymorphisms in Pfmdr1, Pfcrt, and Pfnhe1 genes are associated with reduced in vitro activities of quinine in Plasmodium falciparum isolates from western Kenya. Antimicrob Agents Chemother 2014;58(7):3737–43.

59. Tsumori Y, Ndounga M, Sunahara T, Hayashida N, Inoue M, Nakazawa S, et al. Plasmodium falciparum: differential selection of drug resistance alleles in contiguous urban and peri-urban areas of Brazzaville, Republic of Congo. PLoS One 2011;6(8):e23430.

60. Eshetu T, Berens-Riha N, Fekadu S, Tadesse Z, Gürkov R, Hölscher M, et al. Different mutation patterns of Plasmodium falciparum among patients in Jimma University Hospital, Ethiopia. Malar J 2010;9:226.

61. Andriantsoanirina V, Ratsimbasoa A, Bouchier C, Jahevitra M, Rabearimanana S, Radrianjafy R, et al. Plasmodium falciparum drug resistance in Madagascar: facing the spread of unusual pfdhfr and pfmdr-1 haplotypes and the decrease of dihydroartemisinin susceptibility. Antimicrob Agents Chemother 2009;53(11):4588–97.

62. Nsimba B, Jafari-Guemouri S, Malonga DA, Mouata AM, Kiori J, Louya F, et al. Epidemiology of drug-resistant malaria in Republic of Congo: using molecular evidence for monitoring antimalarial drug resistance combined with assessment of antimalarial drug use. Trop Med Int Health 2005;10(10):1030–7.

63. Molecular markers of resistance to sulphadoxine-pyrimethamine one year after implementation of intermittent preventive treatment of malaria in infants in Mali - PubMed [Internet]. [cited 2025 Sep 26];Available from: https://pubmed.ncbi.nlm.nih.gov/20064223/

64. …, Munyaneza T, Mazarati J, Munguti K, Campagne P, ... Emergence and clonal expansion of in vitro artemisinin-resistant Plasmodium falciparum kelch13 R561H mutant parasites in Rwanda. Nature medicine [Internet] 2020;(Query date: 2025-07-02 12:44:41). Available from: https://www.nature.com/articles/s41591-020-1005-2

65. Alker AP, Mwapasa V, Purfield A, Rogerson SJ, Molyneux ME, Kamwendo DD, et al. Mutations Associated with Sulfadoxine-Pyrimethamine and Chlorproguanil Resistance in Plasmodium falciparum Isolates from Blantyre, Malawi. Antimicrob Agents Chemother 2005;49(9):3919–21.

66. Alruwaili M, Uwimana A, Sethi R, Murindahabi M, Piercefield E, Umulisa N, et al. Peripheral and Placental Prevalence of Sulfadoxine-Pyrimethamine Resistance Markers in Plasmodium falciparum among Pregnant Women in Southern Province, Rwanda. Am J Trop Med Hyg 2023;109(5):1057–62.

67. Amenga-Etego LN, Asoala V, Agongo G, Jacob C, Goncalves S, Awandare GA, et al. Temporal evolution of sulfadoxine-pyrimethamine resistance genotypes and genetic diversity in response to a decade of increased interventions against Plasmodium falciparum in northern Ghana. Malar J 2021;20(1):152.

68. Bakari C, Mandara CI, Madebe RA, Seth MD, Ngasala B, Kamugisha E, et al. Trends of Plasmodium falciparum molecular markers associated with resistance to artemisinins and reduced susceptibility to lumefantrine in Mainland Tanzania from 2016 to 2021. Malar J 2024;23(1):71.

69. Baliraine FN, Nsobya SL, Achan J, Tibenderana JK, Talisuna AO, Greenhouse B, et al. Limited ability of Plasmodium falciparum pfcrt, pfmdr1, and pfnhe1 polymorphisms to predict quinine in vitro sensitivity or clinical effectiveness in Uganda. Antimicrob Agents Chemother 2011;55(2):615–22.

70. Bansal D, Bharti PK, Acharya A, Abdelraheem MH, Patel P, Elmalik A, et al. Molecular surveillance of putative drug resistance markers of antifolate and artemisinin among imported Plasmodium falciparum in Qatar. Pathog Glob Health 2019;113(4):158–66.

71. Bergmann C, van Loon W, Habarugira F, Tacoli C, Jäger JC, Savelsberg D, et al. Increase in Kelch 13 Polymorphisms in Plasmodium falciparum, Southern Rwanda. Emerg Infect Dis 2021;27(1):294–6.

72. Happi CT, Gbotosho GO, Folarin OA, Bolaji OM, Sowunmi A, Kyle DE, et al. Association between mutations in Plasmodium falciparum chloroquine resistance transporter and P. falciparum multidrug resistance 1 genes and in vivo amodiaquine resistance in P. falciparum malaria-infected children in Nigeria. Am J Trop Med Hyg 2006;75(1):155–61.

73. Dokomajilar C, Nsobya S, Greenhouse B, ... Selection of Plasmodium falciparum pfmdr1 Alleles following Therapy with Artemether-Lumefantrine in an Area of Uganda where Malaria Is Highly Endemic. Antimicrobial agents … [Internet] 2006;(Query date: 2025-07-02 12:44:41). Available from: https://journals.asm.org/doi/abs/10.1128/aac.50.5.1893-1895.2006

74. Coulibaly A, Diop MF, Kone A, Dara A, Ouattara A, Mulder N, et al. Genome-wide SNP analysis of Plasmodium falciparum shows differentiation at drug-resistance-associated loci among malaria transmission settings in southern Mali. Front Genet 2022;13:943445.

75. da Silva C, Boene S, Datta D, Rovira-Vallbona E, Aranda-Díaz A, Cisteró P, et al. Targeted and whole-genome sequencing reveal a north-south divide in P. falciparum drug resistance markers and genetic structure in Mozambique. Commun Biol 2023;6(1):619.

76. Selection of pfmdr1 and pfcrt alleles in amodiaquine treatment failure in north-western Burkina Faso - PubMed [Internet]. [cited 2025 Sep 26];Available from: https://pubmed.ncbi.nlm.nih.gov/20060374/

77. Diakite M, Achidi EA, Achonduh O, Craik R, Djimde AA, Evehe MSB, et al. Host candidate gene polymorphisms and clearance of drug-resistant Plasmodium falciparum parasites. Malar J 2011;10:250.

78. Dieng CC, Gonzalez L, Pestana K, Dhikrullahi SB, Amoah LE, Afrane YA, et al. Contrasting Asymptomatic and Drug Resistance Gene Prevalence of Plasmodium falciparum in Ghana: Implications on Seasonal Malaria Chemoprevention. Genes (Basel) 2019;10(7).

79. Doumbo S, Ongoiba OA, Doumtabé D, Dara A, Ouologuem TD, Kayentao K, et al. [Prevalence of Plasmodium falciparum, anemia and molecular markers of chloroquine and sulfadoxine-pyriméthamine resistance in delivered women in Fana, Mali]. Bull Soc Pathol Exot 2013;106(3):188–92.

80. Ebel ER, Reis F, Petrov DA, Beleza S. Historical trends and new surveillance of Plasmodium falciparum drug resistance markers in Angola. Malar J 2021;20(1):175.

81. Ehrhardt S, Eggelte TA, Kaiser S, Adjei L, Burchard GD, Anemana SD, et al. Large-scale surveillance of Plasmodium falciparum crt(K76T) in northern Ghana. Antimicrob Agents Chemother 2007;51(9):3407–9.

82. Esu E, Tacoli C, Gai P, Berens-Riha N, Pritsch M, Loescher T, et al. Prevalence of the Pfdhfr and Pfdhps mutations among asymptomatic pregnant women in Southeast Nigeria. Parasitol Res 2018;117(3):801–7.

83. Efficacy and effectiveness of the combination of sulfadoxine/pyrimethamine and a 3-day course of artesunate for the treatment of uncomplicated falciparum malaria in a refugee settlement in Zambia | Cochrane Library [Internet]. [cited 2025 Jul 2];Available from: https://www.cochranelibrary.com/central/doi/10.1002/central/CN-00502874/full

84. Mbogo GW, Nankoberanyi S, Tukwasibwe S, Baliraine FN, Nsobya SL, Conrad MD, et al. Temporal changes in prevalence of molecular markers mediating antimalarial drug resistance in a high malaria transmission setting in Uganda. Am J Trop Med Hyg 2014;91(1):54–61.

85. Gikunju SW, Agola EL, Ondondo RO, Kinyua J, Kimani F, LaBeaud AD, et al. Prevalence of pfdhfr and pfdhps mutations in Plasmodium falciparum associated with drug resistance among pregnant women receiving IPTp-SP at Msambweni County Referral Hospital, Kwale County, Kenya. Malar J 2020;19(1):190.

86. Gupta H, Galatas B, Chidimatembue A, Huijben S, Cisteró P, Matambisso G, et al. Effect of mass dihydroartemisinin-piperaquine administration in southern Mozambique on the carriage of molecular markers of antimalarial resistance. PLoS One 2020;15(10):e0240174.

87. Hailemeskel E, Menberu T, Shumie G, Behaksra S, Chali W, Keffale M, et al. Prevalence of Plasmodium falciparum Pfcrt and Pfmdr1 alleles in settings with different levels of Plasmodium vivax co-endemicity in Ethiopia. Int J Parasitol Drugs Drug Resist 2019;11:8–12.

88. Happi CT, Gbotosho GO, Folarin OA, Akinboye DO, Yusuf BO, Ebong OO, et al. Polymorphisms in Plasmodium falciparum dhfr and dhps genes and age related in vivo sulfadoxine-pyrimethamine resistance in malaria-infected patients from Nigeria. Acta Trop 2005;95(3):183–93.

89. …, Hetzel M, Laman M, Barry A, Ringwald P, ... Emergence of artemisinin-resistant Plasmodium falciparum with kelch13 C580Y mutations on the island of New Guinea. PLoS … [Internet] 2020;(Query date: 2025-07-02 12:44:41). Available from: https://journals.plos.org/plospathogens/article?id=10.1371/journal.ppat.1009133

90. Heuchert A, Abduselam N, Zeynudin A, Eshetu T, Löscher T, Wieser A, et al. Molecular markers of anti-malarial drug resistance in southwest Ethiopia over time: regional surveillance from 2006 to 2013. Malar J 2015;14:208.

91. Huang B, Wang Q, Deng C, Wang J, Yang T, Huang S, et al. Prevalence of crt and mdr-1 mutations in Plasmodium falciparum isolates from Grande Comore island after withdrawal of chloroquine. Malar J 2016;15(1):414.

92. Humphreys GS, Merinopoulos I, Ahmed J, Whitty CJM, Mutabingwa TK, Sutherland CJ, et al. Amodiaquine and artemether-lumefantrine select distinct alleles of the Plasmodium falciparum mdr1 gene in Tanzanian children treated for uncomplicated malaria. Antimicrob Agents Chemother 2007;51(3):991–7.

93. Ibrahim ML, Gay-Andrieu F, Adehossi E, Lacroix V, Randrianarivelojosia M, Duchemin JB. Field-based evidence for the linkage of pfcrt and pfdhfr drug-resistant malaria genotypes and clinical profiles of severe malaria in Niger. Microbes Infect 2007;9(5):599–604.

94. Igbasi UT, Oyibo WA, Chen JH, Quan H, Omilabu SA, Chen SB, et al. Haplotypes of Chloroquine Resistance Marker Genes Among Uncomplicated Malaria Cases in Lagos, Nigeria. Biochem Genet 2025;

95. Ishengoma DS, Mandara CI, Bakari C, Fola AA, Madebe RA, Seth MD, et al. Evidence of artemisinin partial resistance in northwestern Tanzania: clinical and molecular markers of resistance. Lancet Infect Dis 2024;24(11):1225–33.

96. Jalei AA, Na-Bangchang K, Muhamad P, Chaijaroenkul W. Monitoring antimalarial drug-resistance markers in Somalia. Parasites Hosts Dis 2023;61(1):78–83.

97. Jiang T, Chen J, Fu H, Wu K, Yao Y, Eyi JUM, et al. High prevalence of Pfdhfr-Pfdhps quadruple mutations associated with sulfadoxine-pyrimethamine resistance in Plasmodium falciparum isolates from Bioko Island, Equatorial Guinea. Malar J 2019;18(1):101.

98. Jovel IT, Björkman A, Roper C, Mårtensson A, Ursing J. Unexpected selections of Plasmodium falciparum polymorphisms in previously treatment-naïve areas after monthly presumptive administration of three different anti-malarial drugs in Liberia 1976-78. Malar J 2017;16(1):113.

99. Maniga JN, Akinola SA, Odoki M, Odda J, Adebayo IA. Limited Polymorphism in Plasmodium falciparum Artemisinin Resistance Kelch13-Propeller Gene Among Clinical Isolates from Bushenyi District, Uganda. Infect Drug Resist 2021;14:5153–63.

100. Fola A, Feleke S, Mohammed H, Brhane B, ... Plasmodium falciparum resistant to artemisinin and diagnostics have emerged in Ethiopia. Nature … [Internet] 2023;(Query date: 2025-07-02 12:44:41). Available from: https://www.nature.com/articles/s41564-023-01461-4

101. Hemming-Schroeder E, Umukoro E, Lo E, Fung B, Tomás-Domingo P, Zhou G, et al. Impacts of Antimalarial Drugs on Plasmodium falciparum Drug Resistance Markers, Western Kenya, 2003-2015. Am J Trop Med Hyg 2018;98(3):692–9.

102. Matondo SI, Temba GS, Kavishe AA, Kauki JS, Kalinga A, van Zwetselaar M, et al. High levels of sulphadoxine-pyrimethamine resistance Pfdhfr-Pfdhps quintuple mutations: a cross sectional survey of six regions in Tanzania. Malar J 2014;13:152.

103. Foumane Ngane V, Allico Djaman J, Culeux C, Piette N, Carnevale P, Besnard P, et al. Molecular epidemiology of drug-resistant Plasmodium falciparum in Benguela province, Angola. Malar J 2015;14:113.

104. Talundzic E, Ndiaye Y, Deme A, ... Molecular epidemiology of Plasmodium falciparum kelch13 mutations in Senegal determined by using targeted amplicon deep sequencing.

Antimicrobial agents … [Internet] 2017;(Query date: 2025-07-02 12:44:41). Available from: https://journals.asm.org/doi/abs/10.1128/aac.02116-16

105. Sitali L, Mwenda MC, Miller JM, Bridges DJ, Hawela MB, Hamainza B, et al. Surveillance of molecular markers for antimalarial resistance in Zambia: Polymorphism of Pfkelch 13, Pfmdr1 and Pfdhfr/Pfdhps genes. Acta Trop 2020;212:105704.

106. Straimer J, Gandhi P, Renner K, ... High Prevalence of Plasmodium falciparum K13 Mutations in Rwanda Is Associated With Slow Parasite Clearance After Treatment With Artemether-Lumefantrine. … Journal of infectious … [Internet] 2022;(Query date: 2025-07-02 12:44:41). Available from: https://academic.oup.com/jid/article-abstract/225/8/1411/6314293

107. Beavogui AH, Diawara EY, Cherif MS, Delamou A, Diallo N, Traore A, et al. SELECTION OF PFCRT 76T AND PFMDR1 86Y MUTANT PLASMODIUM FALCIPARUM AFTER TREATMENT OF UNCOMPLICATED MALARIA WITH ARTESUNATE-AMODIAQUINE IN REPUBLIC OF GUINEA. J Parasitol 2021;107(5):778–82.

108. Karema C, Imwong M, Fanello CI, Stepniewska K, Uwimana A, Nakeesathit S, et al. Molecular correlates of high-level antifolate resistance in Rwandan children with Plasmodium falciparum malaria. Antimicrob Agents Chemother 2010;54(1):477–83.

109. Voumbo-Matoumona DF, Akiana J, Madamet M, Kouna LC, Lekana-Douki JB, Pradines B. High prevalence of Plasmodium falciparum antimalarial drug resistance markers in isolates from asymptomatic patients from the Republic of the Congo between 2010 and 2015. J Glob Antimicrob Resist 2018;14:277–83.

110. Olukosi Y, Oyebola M, Ajibaye O, ... Persistence of markers of chloroquine resistance among P. falciparum isolates recovered from two Nigerian communities. MalariaWorld … [Internet] 2014;(Query date: 2025-07-02 12:44:41). Available from: https://pmc.ncbi.nlm.nih.gov/articles/PMC11100369/

111. Somé A, Sorgho H, Zongo I, Bazié T, Nikiéma F, ... Polymorphisms in K13, pfcrt, pfmdr1, pfdhfr, and pfdhps in parasites isolated from symptomatic malaria patients in Burkina Faso. Parasite [Internet] 2016;(Query date: 2025-07-02 12:44:41). Available from: https://pmc.ncbi.nlm.nih.gov/articles/PMC5178381/

112. Dakorah MP, Aninagyei E, Attoh J, Adzakpah G, Tukwarlba I, Acheampong DO. Profiling antimalarial drug-resistant haplotypes in Pfcrt, Pfmdr1, Pfdhps and Pfdhfr genes in Plasmodium falciparum causing malaria in the Central Region of Ghana: a multicentre cross-sectional study. Ther Adv Infect Dis 2025;12:20499361251319665.

113. Nguetse CN, Adegnika AA, Agbenyega T, Ogutu BR, Krishna S, Kremsner PG, et al. Molecular markers of anti-malarial drug resistance in Central, West and East African children with severe malaria. Malar J 2017;16(1):217.

114. Wurtz N, Fall B, Pascual A, Fall M, Baret E, ... Role of Pfmdr1 in In Vitro Plasmodium falciparum Susceptibility to Chloroquine, Quinine, Monodesethylamodiaquine, Mefloquine, Lumefantrine, and …. Antimicrobial agents … [Internet] 2014;(Query date: 2025-07-02 12:44:41). Available from: https://journals.asm.org/doi/abs/10.1128/aac.03494-14

115. Dlamini SV, Beshir K, Sutherland CJ. Markers of anti-malarial drug resistance in Plasmodium falciparum isolates from Swaziland: identification of pfmdr1-86F in natural parasite isolates. Malar J 2010;9:68.

116. Agomo CO, Oyibo WA, Sutherland C, Hallet R, Oguike M. Assessment of Markers of Antimalarial Drug Resistance in Plasmodium falciparum Isolates from Pregnant Women in Lagos, Nigeria. PLoS One 2016;11(1):e0146908.

117. Li J, Chen J, Xie D, Eyi UM, Matesa RA, Obono MMO, et al. Molecular mutation profile of Pfcrt and Pfmdr1 in Plasmodium falciparum isolates from Bioko Island, Equatorial Guinea. Infect Genet Evol 2015;36:552–6.

118. Dentinger CM, Rakotomanga TA, Rakotondrandriana A, Rakotoarisoa A, Rason MA, Moriarty LF, et al. Efficacy of artesunate-amodiaquine and artemether-lumefantrine for uncomplicated Plasmodium falciparum malaria in Madagascar, 2018. Malar J 2021;20(1):432.

119. Prevalence of mutations associated with artemisinin partial resistance and sulfadoxine-pyrimethamine resistance in 13 regions in Tanzania in 2021: a cross-sectional survey - PubMed [Internet]. [cited 2025 Jul 22];Available from: https://pubmed.ncbi.nlm.nih.gov/39159629/

120. Kavishe R, Paulo P, Kaaya R, Kalinga A, ... Surveillance of artemether-lumefantrine associated Plasmodium falciparum multidrug resistance protein-1 gene polymorphisms in Tanzania. Malaria journal [Internet] 2014;(Query date: 2025-07-02 12:44:41). Available from: https://link.springer.com/article/10.1186/1475-2875-13-264

121. Boukoumba FM, Lekana-Douki JB, Matsiegui PB, Moukodoum DN, Adegnika AA, Oyegue-Liabagui SL. High prevalence of genotypes associated with sulfadoxine/pyrimethamine resistance in the rural area of Fougamou, Gabon. J Glob Antimicrob Resist 2021;25:181–6.

122. Ursing J, Kofoed PE, Rodrigues A, Rombo L, Gil JP. Plasmodium falciparum genotypes associated with chloroquine and amodiaquine resistance in Guinea-Bissau. Am J Trop Med Hyg 2007;76(5):844–8.

123. Noranate N, Durand R, Tall A, Marrama L, Spiegel A, ... Rapid Dissemination of Plasmodium falciparum Drug Resistance Despite Strictly Controlled Antimalarial Use. PLoS … [Internet] 2007;(Query date: 2025-07-02 12:44:41). Available from: https://journals.plos.org/plosone/article?id=10.1371/journal.pone.0000139

124. Holmgren G, Björkman A, Gil JP. Amodiaquine resistance is not related to rare findings of pfmdr1 gene amplifications in Kenya. Trop Med Int Health 2006;11(12):1808–12.

125. Narh CA, Ghansah A, Duffy MF, Ruybal-Pesántez S, Onwona CO, Oduro AR, et al. Evolution of Antimalarial Drug Resistance Markers in the Reservoir of Plasmodium falciparum Infections in the Upper East Region of Ghana. J Infect Dis 2020;222(10):1692–701.

126. Gadalla NB, Tavera G, Mu J, Kabyemela ER, Fried M, Duffy PE, et al. Prevalence of Plasmodium falciparum anti-malarial resistance-associated polymorphisms in pfcrt, pfmdr1 and pfnhe1 in Muheza, Tanzania, prior to introduction of artemisinin combination therapy. Malar J 2015;14:129.

127. Mayengue PI, Ndounga M, Davy MM, Tandou N, Ntoumi F. In vivo chloroquine resistance and prevalence of the pfcrt codon 76 mutation in Plasmodium falciparum isolates from the Republic of Congo. Acta Trop 2005;95(3):219–25.

128. Somé AF, Zongo I, Compaoré YD, Sakandé S, Nosten F, Ouédraogo JB, et al. Selection of drug resistance-mediating Plasmodium falciparum genetic polymorphisms by seasonal malaria chemoprevention in Burkina Faso. Antimicrob Agents Chemother 2014;58(7):3660–5.

129. Dorkenoo AM, Warsame M, Ataba E, Hemou M, Yakpa K, Sossou E, et al. Efficacy of artemether-lumefantrine and dihydroartemisinin-piperaquine and prevalence of molecular markers of anti-malarial drug resistance in children in Togo in 2021. Malar J 2024;23(1):92.

130. Hodoameda P, Duah-Quashie NO, Hagan CO, Matrevi S, Abuaku B, Koram K, et al. Plasmodium falciparum genetic factors rather than host factors are likely to drive resistance to ACT in Ghana. Malar J 2020;19(1):255.

131. Moriarty LF, Nkoli PM, Likwela JL, Mulopo PM, Sompwe EM, Rika JM, et al. Therapeutic Efficacy of Artemisinin-Based Combination Therapies in Democratic Republic of the Congo and Investigation of Molecular Markers of Antimalarial Resistance. Am J Trop Med Hyg 2021;105(4):1067–75.

132. Galatas B, Nhamussua L, Candrinho B, Mabote L, Cisteró P, Gupta H, et al. In-Vivo Efficacy of Chloroquine to Clear Asymptomatic Infections in Mozambican Adults: A Randomized, Placebo-controlled Trial with Implications for Elimination Strategies. Sci Rep 2017;7(1):1356.

133. Nawaz F, Nsobya SL, Kiggundu M, Joloba M, Rosenthal PJ. Selection of parasites with diminished drug susceptibility by amodiaquine-containing antimalarial regimens in Uganda. J Infect Dis 2009;200(11):1650–7.

134. Shah M, Kariuki S, Vanden Eng J, Blackstock AJ, Garner K, Gatei W, et al. Effect of transmission reduction by insecticide-treated bednets (ITNs) on antimalarial drug resistance in western Kenya. PLoS One 2011;6(11):e26746.

135. Beavogui AH, Camara A, Delamou A, Diallo MS, Doumbouya A, Kourouma K, et al. Efficacy and safety of artesunate-amodiaquine and artemether-lumefantrine and prevalence of molecular markers associated with resistance, Guinea: an open-label two-arm randomised controlled trial. Malar J 2020;19(1):223.

136. Sitali L, Mwenda MC, Miller JM, Bridges DJ, Hawela MB, Chizema-Kawesha E, et al. En-route to the "elimination" of genotypic chloroquine resistance in Western and Southern Zambia, 14 years after chloroquine withdrawal. Malar J 2019;18(1):391.

137. Bell DJ, Nyirongo SK, Mukaka M, Zijlstra EE, Plowe CV, Molyneux ME, et al. Sulfadoxine-pyrimethamine-based combinations for malaria: a randomised blinded trial to compare efficacy, safety and selection of resistance in Malawi. PLoS One 2008;3(2):e1578.

138. Sisowath C, Petersen I, Veiga MI, Mårtensson A, Premji Z, Björkman A, et al. In vivo selection of Plasmodium falciparum parasites carrying the chloroquine-susceptible pfcrt K76 allele after treatment with artemether-lumefantrine in Africa. J Infect Dis 2009;199(5):750–7.

139. Ursing J, Kofoed PE, Rodrigues A, Blessborn D, Thoft-Nielsen R, Björkman A, et al. Similar efficacy and tolerability of double-dose chloroquine and artemether-lumefantrine for treatment of Plasmodium falciparum infection in Guinea-Bissau: a randomized trial. J Infect Dis 2011;203(1):109–16.

140. Mbacham WF, Evehe MSB, Netongo PM, Ateh IA, Mimche PN, Ajua A, et al. Efficacy of amodiaquine, sulphadoxine-pyrimethamine and their combination for the treatment of uncomplicated Plasmodium falciparum malaria in children in Cameroon at the time of policy change to artemisinin-based combination therapy. Malar J 2010;9:34.

141. Ako BA, Offianan AT, Johansson M, Penali LK, Nguetta SPA, Sibley CH. Molecular analysis of markers associated with chloroquine and sulfadoxine/pyrimethamine resistance in Plasmodium falciparum malaria parasites from southeastern Côte-d'Ivoire by the time of Artemisinin-based Combination Therapy adoption in 2005. Infect Drug Resist 2012;5:113–20.

142. Jm NN, Dp MM, Np M, R NNE, Mk BA. Increased Prevalence of Mutant Allele Pfdhps 437G and Pfdhfr Triple Mutation in Plasmodium falciparum Isolates from a Rural Area of Gabon, Three Years after the Change of Malaria Treatment Policy. Malaria research and treatment [Internet] 2016 [cited 2025 Sep 26];2016. Available from: https://pubmed.ncbi.nlm.nih.gov/27190671/

143. Dagnogo O, Ako AAB, Dago DN, Kouman KBA, Coulibaly ND, Bla KB, et al. Prevalence and genetic diversity of polymorphisms in pfcrt, pfdhfr-ts and pfk13 propeller genes of Plasmodium falciparum in southern Côte d'Ivoire. Malariaworld J 2025;16:1.

144. Millogo KS, Zabré A, Sondo P, Kaboré B, Kouevi AFC, Compaoré EW, et al. Seasonal malaria chemoprevention and mutations in Pfdhfr and Pfdhps genes in children in the health district of Nanoro, Burkina Faso. Malariaworld J 2025;16:5.

145. Tchuenkam PVK, Ngum LN, Ali IM, Chedjou JPK, Nji AM, Netongo PM, et al. Plasmodium falciparum dhps and dhfr markers of resistance to sulfadoxine-pyrimethamine five years (2016-2020) after the implementation of seasonal malaria chemoprevention in Cameroon. Wellcome Open Res 2024;9:323.

146. Muhammad R, Nock I, Ndams I, ... Distribution of Pfmdr1 and Pfcrt chloroquine drug resistance alleles in north-western Nigeria. MalariaWorld … [Internet] 2017;(Query date: 2025-07-02 12:44:41). Available from: https://pmc.ncbi.nlm.nih.gov/articles/PMC8415075/

147. Soniran OT, Idowu OA, Ogundapo SS. Factors associated with high prevalence of PfCRT K76T mutation in Plasmodium falciparum isolates in a rural and urban community of Ogun State, Nigeria. Malariaworld J 2017;8:13.

148. Aninagyei E, Duedu KO, Rufai T, Tetteh CD, Chandi MG, Ampomah P, et al. Characterization of putative drug resistant biomarkers in Plasmodium falciparum isolated from Ghanaian blood donors. BMC Infect Dis 2020;20(1):533.

149. Salissou A, Zamanka H, Biyghe Binze B, Rivière T, Tichit M, Ibrahim ML, et al. Low Prevalence of Pfcrt Resistance Alleles among Patients with Uncomplicated Falciparum Malaria in Niger Six Years after Chloroquine Withdrawal. Malar Res Treat 2014;2014:614190.

150. Comparative efficacy of uncontrolled and controlled intermittent preventive treatment during pregnancy (IPTp) with combined use of LLTNs in high resistance area to sulfadoxine-pyrimethamine in Côte d'Ivoire - PubMed [Internet]. [cited 2025 Sep 26];Available from: https://pubmed.ncbi.nlm.nih.gov/22442633/

151. At O, Lk P, M C, N T, A A, E A, et al. Comparative efficacy of uncontrolled and controlled intermittent preventive treatment during pregnancy (IPTp) with combined use of LLTNs in high resistance area to sulfadoxine-pyrimethamine in Côte d'Ivoire. Infection and drug resistance [Internet] 2012 [cited 2025 Sep 26];5. Available from: https://pubmed.ncbi.nlm.nih.gov/22442633/

152. Assessment of Plasmodium falciparum anti-malarial drug resistance markers in pfk13-propeller, pfcrt and pfmdr1 genes in isolates from treatment failure patients in Democratic Republic of Congo, 2018–2019 | Malaria Journal | Full Text [Internet]. [cited 2025 Sep 26];Available from: https://malariajournal.biomedcentral.com/articles/10.1186/s12936-021-03636-y

153. Adam R, Mukhtar MM, Abubakar UF, Damudi HA, Muhammad A, Ibrahim SS. Polymorphism Analysis of pfmdr1 and pfcrt from Plasmodium falciparum Isolates in Northwestern Nigeria Revealed the Major Markers Associated with Antimalarial Resistance. Diseases 2021;9(1):6.

154. Bwire GM, Ngasala B, Mikomangwa WP, Kilonzi M, Kamuhabwa AAR. Detection of mutations associated with artemisinin resistance at k13-propeller gene and a near complete

return of chloroquine susceptible falciparum malaria in Southeast of Tanzania. Sci Rep 2020;10(1):3500.

155. Figueiredo P, Benchimol C, Lopes D, Bernardino L, do Rosário VE, Varandas L, et al. Prevalence of pfmdr1, pfcrt, pfdhfr and pfdhps mutations associated with drug resistance, in Luanda, Angola. Malar J 2008;7:236.

156. Chebore W, Zhou Z, Westercamp N, Otieno K, Shi YP, Sergent SB, et al. Assessment of molecular markers of anti-malarial drug resistance among children participating in a therapeutic efficacy study in western Kenya. Malar J 2020;19(1):291.

157. Duah NO, Matrevi SA, de Souza DK, Binnah DD, Tamakloe MM, Opoku VS, et al. Increased pfmdr1 gene copy number and the decline in pfcrt and pfmdr1 resistance alleles in Ghanaian Plasmodium falciparum isolates after the change of anti-malarial drug treatment policy. Malar J 2013;12:377.

158. Tuedom AGB, Sarah-Matio EM, Moukoko CEE, Feufack-Donfack BL, Maffo CN, Bayibeki AN, et al. Antimalarial drug resistance in the Central and Adamawa regions of Cameroon: Prevalence of mutations in P. falciparum crt, Pfmdr1, Pfdhfr and Pfdhps genes. PLoS One 2021;16(8):e0256343.

159. Identification of Mutations in Antimalarial Resistance Gene Kelch13 from Plasmodium falciparum Isolates in Kano, Nigeria - PMC [Internet]. [cited 2025 Sep 26];Available from: https://pmc.ncbi.nlm.nih.gov/articles/PMC7345473/

160. Zhou Z, Gimnig JE, Sergent SB, Liu Y, Abong'o B, Otieno K, et al. Temporal trends in molecular markers of drug resistance in Plasmodium falciparum in human blood and profiles of corresponding resistant markers in mosquito oocysts in Asembo, western Kenya. Malar J 2022;21(1):265.

161. Idowu AO, Oyibo WA, Bhattacharyya S, Khubbar M, Mendie UE, Bumah VV, et al. Rare mutations in Pfmdr1 gene of Plasmodium falciparum detected in clinical isolates from patients treated with anti-malarial drug in Nigeria. Malaria Journal 2019;18(1):319.

162. Eyase FL, Akala HM, Ingasia L, Cheruiyot A, Omondi A, Okudo C, et al. The role of Pfmdr1 and Pfcrt in changing chloroquine, amodiaquine, mefloquine and lumefantrine susceptibility in western-Kenya P. falciparum samples during 2008-2011. PLoS One 2013;8(5):e64299.

163. Baina MT, Djontu JC, Mbama Ntabi JD, Mfoutou Mapanguy CC, Lissom A, Vouvoungui CJ, et al. Polymorphisms in the Pfcrt, Pfmdr1, and Pfk13 genes of Plasmodium falciparum isolates from southern Brazzaville, Republic of Congo. Sci Rep 2024;14(1):27988.

164. L'Episcopia M, Doderer-Lang C, Perrotti E, Priuli GB, Cavallari S, Guidetti C, et al. Polymorphism analysis of drug resistance markers in Plasmodium falciparum isolates from Benin. Acta Trop 2023;245:106975.

165. Musyoka KB, Kiiru JN, Aluvaala E, Omondi P, Chege WK, Judah T, et al. Prevalence of mutations in *Plasmodium falciparum* genes associated with resistance to different antimalarial drugs in Nyando, Kisumu County in Kenya. Infection, Genetics and Evolution [Internet] 2020 [cited 2025 Jul 22];78:104121. Available from: https://www.sciencedirect.com/science/article/pii/S1567134819303478

166. Drug resistance profile and clonality of Plasmodium falciparum parasites in Cape Verde: the 2017 malaria outbreak | Malaria Journal | Full Text [Internet]. [cited 2025 Sep 26];Available from: https://malariajournal.biomedcentral.com/articles/10.1186/s12936-021-03708-z

167. Restrepo E, Carmona-Fonseca J, Maestre A. Plasmodium falciparum: high frequency of pfcrt point mutations and emergence of new mutant haplotypes in Colombia. Biomedica 2008;28(4):523–30.

168. Chenet SM, Okoth SA, Kelley J, Lucchi N, Huber CS, Vreden S, et al. Molecular Profile of Malaria Drug Resistance Markers of Plasmodium falciparum in Suriname. Antimicrob Agents Chemother 2017;61(7).

169. Itoh M, Negreiros do Valle S, Farias S, Holanda de Souza TM, Rachid Viana GM, Lucchi N, et al. Efficacy of Artemether-Lumefantrine for Uncomplicated Plasmodium falciparum Malaria in Cruzeiro do Sul, Brazil, 2016. Am J Trop Med Hyg 2018;98(1):88–94.

170. Pelleau S, Moss EL, Dhingra SK, Volney B, Casteras J, Gabryszewski SJ, et al. Adaptive evolution of malaria parasites in French Guiana: Reversal of chloroquine resistance by acquisition of a mutation in pfcrt. Proc Natl Acad Sci U S A 2015;112(37):11672–7.

171. de Abreu-Fernandes R, de Queiroz LT, Almeida-de-Oliveira NK, de Lavigne Mello AR, de Aguiar Barros J, Pratt-Riccio LR, et al. Tracking Drug Resistance in Plasmodium falciparum: Genetic Diversity of Key Resistance Markers in Brazilian Malaria Hotspots. Int J Mol Sci 2025;26(13):5977.

172. Ñacata I, Early AM, Boboy J, Neafsey DE, Sáenz FE. Tracking Plasmodium falciparum antimalarial resistance markers during a malaria pre-elimination period in the Pacific coast of South America. Sci Rep 2025;15(1):25376.

173. Griffing S, Syphard L, Sridaran S, ... pfmdr1 Amplification and Fixation of pfcrt Chloroquine Resistance Alleles in Plasmodium falciparum in Venezuela. Antimicrobial agents … [Internet] 2010;(Query date: 2025-07-02 12:44:41). Available from: https://journals.asm.org/doi/abs/10.1128/aac.01243-09

174. Torres R, Banegas E, Mendoza M, ... Efficacy of chloroquine for the treatment of uncomplicated Plasmodium falciparum malaria in Honduras. … American journal of … [Internet] 2013;(Query date: 2025-07-02 12:44:41). Available from: https://pmc.ncbi.nlm.nih.gov/articles/PMC3752747/

175. Restrepo-Pineda E, Arango E, Maestre A, Do Rosário VE, Cravo P. Studies on antimalarial drug susceptibility in Colombia, in relation to Pfmdr1 and Pfcrt. Parasitology 2008;135(5):547–53.

176. Baldeviano GC, Okoth SA, Arrospide N, Gonzalez RV, Sánchez JF, Macedo S, et al. Molecular Epidemiology of Plasmodium falciparum Malaria Outbreak, Tumbes, Peru, 2010-2012. Emerg Infect Dis 2015;21(5):797–803.

177. Gama B, Oliveira N de, Zalis M, Souza J de, ... Chloroquine and sulphadoxine-pyrimethamine sensitivity of Plasmodium falciparum parasites in a Brazilian endemic area. Malaria journal [Internet] 2009;(Query date: 2025-07-02 12:44:41). Available from: https://link.springer.com/article/10.1186/1475-2875-8-156

178. Bamaga OAA, Mahdy MAK, Lim YAL. Survey of chloroquine-resistant mutations in the Plasmodium falciparum pfcrt and pfmdr-1 genes in Hadhramout, Yemen. Acta Trop 2015;149:59–63.

179. Khan AQ, Pernaute-Lau L, Khattak AA, Luijcx S, Aydin-Schmidt B, Hussain M, et al. Surveillance of genetic markers associated with Plasmodium falciparum resistance to artemisinin-based combination therapy in Pakistan, 2018-2019. Malar J 2020;19(1):206.

180. Yaqoob A, Khattak A, Nadeem M, Fatima H, ... Prevalence of molecular markers of sulfadoxine–pyrimethamine and artemisinin resistance in Plasmodium falciparum from Pakistan. Malaria journal [Internet] 2018;(Query date: 2025-07-02 12:44:41). Available from: https://link.springer.com/article/10.1186/s12936-018-2620-y

181. Alareqi LMQ, Mahdy MAK, Lau YL, Fong MY, Abdul-Ghani R, Mahmud R. Molecular markers associated with resistance to commonly used antimalarial drugs among Plasmodium falciparum isolates from a malaria-endemic area in Taiz governorate-Yemen during the transmission season. Acta Trop 2016;162:174–9.

182. Warsame M, Hassan AM, Hassan AH, Jibril AM, Khim N, Arale AM, et al. High therapeutic efficacy of artemether-lumefantrine and dihydroartemisinin-piperaquine for the treatment of uncomplicated falciparum malaria in Somalia. Malar J 2019;18(1):231.

183. Hussien M, Abdel Hamid MM, Elamin EA, Hassan AO, Elaagip AH, Salama AHA, et al. Antimalarial drug resistance molecular makers of Plasmodium falciparum isolates from Sudan during 2015-2017. PLoS One 2020;15(8):e0235401.

184. Khattak AA, Venkatesan M, Jacob CG, Artimovich EM, Nadeem MF, Nighat F, et al. A comprehensive survey of polymorphisms conferring anti-malarial resistance in Plasmodium falciparum across Pakistan. Malar J 2013;12:300.

185. Mohamed A, Hussien M, Mohamed A, Suliman A, ... Assessment of Plasmodium falciparum drug resistance molecular markers from the Blue Nile State, Southeast Sudan. Malaria Journal [Internet] 2020;(Query date: 2025-07-02 12:44:41). Available from: https://link.springer.com/article/10.1186/s12936-020-03165-0

186. Mohamed NS, Abdelbagi H, Osman HA, Ahmed AE, Yousif AM, Edris YB, et al. A snapshot of Plasmodium falciparum malaria drug resistance markers in Sudan: a pilot study. BMC Res Notes 2020;13(1):512.

187. Al-Mekhlafi HM, Madkhali AM, Abdulhaq AA, Atroosh WM, Ghzwani AH, Zain KA, et al. Polymorphism analysis of pfmdr1 gene in Plasmodium falciparum isolates 11 years post-adoption of artemisinin-based combination therapy in Saudi Arabia. Sci Rep 2022;12(1):517.

188. Dajem SB, Al-Qahtani A. Analysis of gene mutations involved in chloroquine resistance in Plasmodium falciparum parasites isolated from patients in the southwest of Saudi Arabia. Annals of Saudi medicine [Internet] 2010;(Query date: 2025-07-02 12:44:41). Available from: https://www.annsaudimed.net/doi/abs/10.4103/0256-4947.62826

189. Dajem S, Al-Farsi H, ... Distribution of drug resistance genotypes in Plasmodium falciparum in an area of limited parasite diversity in Saudi Arabia. … American journal of … [Internet] 2012;(Query date: 2025-07-02 12:44:41). Available from: https://pmc.ncbi.nlm.nih.gov/articles/PMC3335680/

190. Jalousian F, Dalimi A, Samiee SM, Ghaffarifar F, Soleymanloo F, Naghizadeh R. Mutation in pfmdr1 gene in chloroquine-resistant Plasmodium falciparum isolates, Southeast Iran. Int J Infect Dis 2008;12(6):630–4.

191. Rana R, Khan N, Sandeepta S, Pati S, Das A, Bal M, et al. Molecular surveillance of anti-malarial drug resistance genes in Plasmodium falciparum isolates in Odisha, India. Malar J 2022;21(1):394.

192. Khammanee T, Sawangjaroen N, Buncherd H, Tun AW, Thanapongpichat S. Molecular Surveillance of Pfkelch13 and Pfmdr1 Mutations in Plasmodium falciparum Isolates from Southern Thailand. Korean J Parasitol 2019;57(4):369–77.

193. Lubis IND, Wijaya H, Lubis M, Lubis CP, Beshir KB, Sutherland CJ. Plasmodium falciparum Isolates Carrying pfk13 Polymorphisms Harbor the SVMNT Allele of pfcrt in Northwestern Indonesia. Antimicrob Agents Chemother 2020;64(8).

194. Imwong M, Dhorda M, Myo Tun K, Thu AM, Phyo AP, Proux S, et al. Molecular epidemiology of resistance to antimalarial drugs in the Greater Mekong subregion: an observational study. Lancet Infect Dis 2020;20(12):1470–80.

195. Valecha N, Srivastava P, Mohanty SS, Mittra P, Sharma SK, Tyagi PK, et al. Therapeutic efficacy of artemether-lumefantrine in uncomplicated falciparum malaria in India. Malar J 2009;8:107.

196. Saha P, Guha SK, Das S, Mullick S, Ganguly S, Biswas A, et al. Comparative efficacies of artemisinin combination therapies in Plasmodium falciparum malaria and polymorphism of pfATPase6, pfcrt, pfdhfr, and pfdhps genes in tea gardens of Jalpaiguri District, India. Antimicrob Agents Chemother 2012;56(5):2511–7.

197. Rahmasari FV, Asih PBS, Rozi IE, Wangsamuda S, Risandi R, Dewayanti FK, et al. Evolution of genetic markers for drug resistance after the introduction of dihydroartemisinin-piperaquine as first-line anti-malarial treatment for uncomplicated falciparum malaria in Indonesia. Malar J 2023;22(1):231.

198. Srimuang K, Miotto O, Lim P, Fairhurst RM, Kwiatkowski DP, Woodrow CJ, et al. Analysis of anti-malarial resistance markers in pfmdr1 and pfcrt across Southeast Asia in the Tracking Resistance to Artemisinin Collaboration. Malar J 2016;15(1):541.

199. Leang R, Taylor WRJ, Bouth DM, Song L, Tarning J, Char MC, et al. Evidence of Plasmodium falciparum Malaria Multidrug Resistance to Artemisinin and Piperaquine in Western Cambodia: Dihydroartemisinin-Piperaquine Open-Label Multicenter Clinical Assessment. Antimicrob Agents Chemother 2015;59(8):4719–26.

200. Khim N, Bouchier C, Ekala MT, Incardona S, Lim P, Legrand E, et al. Countrywide survey shows very high prevalence of Plasmodium falciparum multilocus resistance genotypes in Cambodia. Antimicrob Agents Chemother 2005;49(8):3147–52.

201. Tun KM, Jeeyapant A, Myint AH, Kyaw ZT, Dhorda M, Mukaka M, et al. Effectiveness and safety of 3 and 5 day courses of artemether-lumefantrine for the treatment of uncomplicated falciparum malaria in an area of emerging artemisinin resistance in Myanmar. Malar J 2018;17(1):258.

202. de Almeida A, Arez AP, Cravo PV, do Rosário VE. Analysis of genetic mutations associated with anti-malarial drug resistance in Plasmodium falciparum from the Democratic Republic of East Timor. Malar J 2009;8:59.

203. Ramani S, Parija SC, Mandal J, Hamide A, Bhat V. Detection of chloroquine and artemisinin resistance molecular markers in Plasmodium falciparum: A hospital based study. Trop Parasitol 2016;6(1):69–77.

204. Patel P, Bharti P, Bansal D, Ali N, Raman R, ... Prevalence of mutations linked to antimalarial resistance in Plasmodium falciparum from Chhattisgarh, Central India: A malaria elimination point of view. Scientific reports [Internet] 2017;(Query date: 2025-07-02 12:44:41). Available from: https://www.nature.com/articles/s41598-017-16866-5

205. Muhamad P, Phompradit P, Chaijaroenkul W, Na-Bangchang K. Distribution patterns of molecular markers of antimalarial drug resistance in Plasmodium falciparum isolates on the Thai-Myanmar border during the periods of 1993-1998 and 2002-2008. BMC Genomics 2024;25(1):269.

206. Win AA, Imwong M, Kyaw MP, Woodrow CJ, Chotivanich K, Hanboonkunupakarn B, et al. K13 mutations and pfmdr1 copy number variation in Plasmodium falciparum malaria in Myanmar. Malar J 2016;15:110.

207. Chaijaroenkul W, Ward S, Mungthin M, Johnson D, ... Sequence and gene expression of chloroquine resistance transporter (pfcrt) in the association of in vitro drugs resistance of

Plasmodium falciparum. Malaria Journal [Internet] 2011;(Query date: 2025-07-02 12:44:41). Available from: https://link.springer.com/article/10.1186/1475-2875-10-42

208. Veiga MI, Ferreira PE, Jörnhagen L, Malmberg M, Kone A, Schmidt BA, et al. Novel polymorphisms in Plasmodium falciparum ABC transporter genes are associated with major ACT antimalarial drug resistance. PLoS One 2011;6(5):e20212.

209. Antony HA, Das S, Parija SC, Padhi S. Sequence analysis of pfcrt and pfmdr1 genes and its association with chloroquine resistance in Southeast Indian Plasmodium falciparum isolates. Genom Data 2016;8:85–90.

210. Wu Y, Soe MT, Aung PL, Zhao L, Zeng W, Menezes L, et al. Efficacy of artemether-lumefantrine for treating uncomplicated Plasmodium falciparum cases and molecular surveillance of drug resistance genes in Western Myanmar. Malar J 2020;19(1):304.

211. Duan M, Bai Y, Deng S, Ruan Y, Zeng W, Li X, et al. Different In Vitro Drug Susceptibility Profile of Plasmodium falciparum Isolates from Two Adjacent Areas of Northeast Myanmar and Molecular Markers for Drug Resistance. Trop Med Infect Dis 2022;7(12).

212. Singh A, Singh MP, Ali NA, Poriya R, Rajvanshi H, Nisar S, et al. Assessment of Plasmodium falciparum drug resistance associated molecular markers in Mandla, Madhya Pradesh, India. Malar J 2023;22(1):375.

213. Guerra AP, Olivera MJ, Cortés LJ, Chenet SM, Macedo de Oliveira A, Lucchi NW. Molecular surveillance for anti-malarial drug resistance and genetic diversity of Plasmodium falciparum after chloroquine and sulfadoxine-pyrimethamine withdrawal in Quibdo, Colombia, 2018. Malar J 2022;21(1):306.

214. Han ZY. A Cross-Sectional Survey of Drug-Resistance Polymorphisms in Plasmodium falciparum K13, Plasmepsin 2 and Pfmdr-1 in Sentinel Sites in Myanmar. 2021 [cited 2025 Sep 26];Available from: https://hdl.handle.net/10161/23148

215. Agrawal S, Moser KA, Morton L, Cummings MP, Parihar A, Dwivedi A, et al. Association of a Novel Mutation in the Plasmodium falciparum Chloroquine Resistance Transporter With Decreased Piperaquine Sensitivity. J Infect Dis 2017;216(4):468–76.

216. Kunasol C, Dondorp AM, Batty EM, Nakhonsri V, Sinjanakhom P, Day NPJ, et al. Comparative analysis of targeted next-generation sequencing for Plasmodium falciparum drug resistance markers. Sci Rep 2022;12(1):5563.

217. Lê HG, Naw H, Kang JM, Võ TC, Myint MK, Htun ZT, et al. Molecular Profiles of Multiple Antimalarial Drug Resistance Markers in Plasmodium falciparum and Plasmodium vivax in the Mandalay Region, Myanmar. Microorganisms 2022;10(10).

218. Kojom Foko LP, Narang G, Jakhan J, Tamang S, Moun A, Singh V. Nationwide spatiotemporal drug resistance genetic profiling from over three decades in Indian Plasmodium falciparum and Plasmodium vivax isolates. Malar J 2023;22(1):236.

219. Buppan P, Seethamchai S, Kuamsab N, Harnyuttanakorn P, Putaporntip C, Jongwutiwes S. Multiple Novel Mutations in Plasmodium falciparum Chloroquine Resistance Transporter Gene during Implementation of Artemisinin Combination Therapy in Thailand. Am J Trop Med Hyg 2018;99(4):987–94.

220. He Y, Campino S, Diez Benavente E, Warhurst DC, Beshir KB, Lubis I, et al. Artemisinin resistance-associated markers in Plasmodium falciparum parasites from the China-Myanmar border: predicted structural stability of K13 propeller variants detected in a low-prevalence area. PLoS One 2019;14(3):e0213686.

221. Das S, Tripathy S, Chattopadhayay S, Das B, Kar Mahapatra S, Hati AK, et al. Progressive increase in point mutations associates chloroquine resistance: Even after withdrawal of chloroquine use in India. Int J Parasitol Drugs Drug Resist 2017;7(3):251–61.

222. Patgiri SJ, Sarma K, Sarmah N, Bhattacharyya N, Sarma DK, Nirmolia T, et al. Characterization of drug resistance and genetic diversity of Plasmodium falciparum parasites from Tripura, Northeast India. Sci Rep 2019;9(1):13704.

223. Srisutham S, Madmanee W, Kouhathong J, Sutawong K, Tripura R, Peto TJ, et al. Ten-year persistence and evolution of Plasmodium falciparum antifolate and anti-sulfonamide resistance markers pfdhfr and pfdhps in three Asian countries. PLoS One 2022;17(12):e0278928.

224. Sugaram R, Suwannasin K, Kunasol C, Mathema VB, Day NPJ, Sudathip P, et al. Molecular characterization of Plasmodium falciparum antifolate resistance markers in Thailand between 2008 and 2016. Malar J 2020;19(1):107.

225. Muhamad P, Phompradit P, Sornjai W, Maensathian T, Chaijaroenkul W, Rueangweerayut R, et al. Polymorphisms of molecular markers of antimalarial drug resistance and relationship with artesunate-mefloquine combination therapy in patients with uncomplicated Plasmodium falciparum malaria in Thailand. Am J Trop Med Hyg 2011;85(3):568–72.

226. Veiga M, Ferreira P, Malmberg M, ... pfmdr1 Amplification Is Related to Increased Plasmodium falciparum In Vitro Sensitivity to the Bisquinoline Piperaquine. Antimicrobial agents … [Internet] 2012;(Query date: 2025-07-02 12:44:41). Available from: https://journals.asm.org/doi/abs/10.1128/aac.06350-11

227. Significant Divergence in Sensitivity to Antimalarial Drugs between Neighboring Plasmodium falciparum Populations along the Eastern Border of Myanmar - PMC [Internet]. [cited 2025 Sep 26];Available from: https://pmc.ncbi.nlm.nih.gov/articles/PMC5278693/

228. Four years' monitoring of in vitro sensitivity and candidate molecular markers of resistance of Plasmodium falciparum to artesunate-mefloquine combination in the Thai-Myanmar border | Malaria Journal | Full Text [Internet]. [cited 2025 Sep 26];Available from: https://malariajournal.biomedcentral.com/articles/10.1186/1475-2875-13-23

229. Chaijaroenkul W, Wisedpanichkij R, Na-Bangchang K. Monitoring of in vitro susceptibilities and molecular markers of resistance of Plasmodium falciparum isolates from Thai-Myanmar border to chloroquine, quinine, mefloquine and artesunate. Acta Trop 2010;113(2):190–4.

230. Molecular and pharmacological determinants of the therapeutic response to artemether-lumefantrine in multidrug-resistant Plasmodium falciparum malaria - PubMed [Internet]. [cited 2025 Sep 26];Available from: https://pubmed.ncbi.nlm.nih.gov/16652314/

231. Amaratunga C, Lim P, Suon S, Sreng S, ... Dihydroartemisinin–piperaquine resistance in Plasmodium falciparum malaria in Cambodia: a multisite prospective cohort study. The Lancet infectious … [Internet] 2016;(Query date: 2025-07-02 12:44:41). Available from: https://www.thelancet.com/journals/laninf/article/PIIS1473-3099(15)00487-9/abstract

232. Chhibber-Goel J, Sharma A. Profiles of Kelch mutations in Plasmodium falciparum across South Asia and their implications for tracking drug resistance. … for Parasitology: Drugs and Drug Resistance [Internet] 2019;(Query date: 2025-07-02 12:44:41). Available from: https://www.sciencedirect.com/science/article/pii/S2211320719300922

233. Duru V, Khim N, Leang R, Kim S, Domergue A, Kloeung N, et al. Plasmodium falciparum dihydroartemisinin-piperaquine failures in Cambodia are associated with mutant K13 parasites presenting high survival rates in novel piperaquine in vitro assays: retrospective and prospective investigations. BMC Med 2015;13:305.

234. Dutta S, Krishna S, Vishwakarma AK, Mishra S, Khandai S, Goswami D, et al. Therapeutic efficacy of artemether-lumefantrine in North-Eastern states of India and prevalence of drug resistance-associated molecular markers. Malar J 2025;24(1):106.

235. Ariey F, Witkowski B, Amaratunga C, Beghain J, Langlois AC, Khim N, et al. A molecular marker of artemisinin-resistant Plasmodium falciparum malaria. Nature 2014;505(7481):50–5.

236. Gupta P, Singh R, Khan H, Raza A, Yadavendu V, Bhatt RM, et al. Genetic profiling of the Plasmodium falciparum population using antigenic molecular markers. ScientificWorldJournal 2014;2014:140867.

237. Huang F, Tang L, Yang H, Zhou S, Sun X, Liu H. Therapeutic efficacy of artesunate in the treatment of uncomplicated Plasmodium falciparum malaria and anti-malarial, drug-resistance marker polymorphisms in populations near the China-Myanmar border. Malar J 2012;11:278.

238. Kuesap J, Suphakhonchuwong N, Kalawong L, Khumchum N. Molecular Markers for Sulfadoxine/Pyrimethamine and Chloroquine Resistance in Plasmodium falciparum in Thailand. Korean J Parasitol 2022;60(2):109–16.

239. …, Lallawmzuala K, Hlimpuia L, Nina PB, ... Epidemiology of malaria and chloroquine resistance in Mizoram, northeastern India, a malaria-endemic region bordering Myanmar.

Malaria journal [Internet] 2020;(Query date: 2025-07-02 12:44:41). Available from: https://link.springer.com/article/10.1186/s12936-020-03170-3

240. Liu H, Xu JW, Deng DW, Wang HY, Nie RH, Yin YJ, et al. Dihydroartemisinin-piperaquine efficacy in Plasmodium falciparum treatment and prevalence of drug-resistant molecular markers along China-Myanmar border in 2014-2023. J Glob Antimicrob Resist 2023;35:271–8.

241. …, Vanachayangkul P, Lertsethtakarn P, Gosi P, ... Plasmodium falciparum phenotypic and genotypic resistance profile during the emergence of Piperaquine resistance in Northeastern Thailand. Scientific Reports [Internet] 2021;(Query date: 2025-07-02 12:44:41). Available from: https://www.nature.com/articles/s41598-021-92735-6

242. Wang Z, Wang Y, Cabrera M, Zhang Y, Gupta B, Wu Y, et al. Artemisinin resistance at the China-Myanmar border and association with mutations in the K13 propeller gene. Antimicrob Agents Chemother 2015;59(11):6952–9.

243. Wang X, Mu J, Li G, Chen P, Guo X, Fu L, et al. Decreased prevalence of the Plasmodium falciparum chloroquine resistance transporter 76T marker associated with cessation of chloroquine use against P. falciparum malaria in Hainan, People's Republic of China. Am J Trop Med Hyg 2005;72(4):410–4.

244. Anvikar A, Sharma B, Sharma S, ... In vitro assessment of drug resistance in Plasmodium falciparum in five States of India. Indian Journal of … [Internet] 2012;(Query date: 2025-07-02 12:44:41). Available from: https://journals.lww.com/ijmr/fulltext/2012/35040/in_vitro_assessment_of_drug_resistance_in.8.aspx

245. Availability and quality of anti-malarials among private sector outlets in Myanmar in 2012: results from a large, community-based, cross-sectional survey before a large-scale intervention | Malaria Journal [Internet]. [cited 2025 Nov 7];Available from: https://link.springer.com/article/10.1186/s12936-015-0778-0

246. Imwong M, Suwannasin K, Srisutham S, Vongpromek R, Promnarate C, Saejeng A, et al. Evolution of Multidrug Resistance in Plasmodium falciparum: a Longitudinal Study of Genetic Resistance Markers in the Greater Mekong Subregion. Antimicrob Agents Chemother 2021;65(12):e0112121.

247. Tavul L, Hetzel MW, Teliki A, Walsh D, Kiniboro B, Rare L, et al. Efficacy of artemether-lumefantrine and dihydroartemisinin-piperaquine for the treatment of uncomplicated malaria in Papua New Guinea. Malar J 2018;17(1):350.

248. Alker AP, Lim P, Sem R, Shah NK, Yi P, Bouth DM, et al. Pfmdr1 and in vivo resistance to artesunate-mefloquine in falciparum malaria on the Cambodian-Thai border. Am J Trop Med Hyg 2007;76(4):641–7.

249. Huang F, Takala-Harrison S, Jacob CG, Liu H, Sun X, Yang H, et al. A Single Mutation in K13 Predominates in Southern China and Is Associated With Delayed Clearance of

Plasmodium falciparum Following Artemisinin Treatment. J Infect Dis 2015;212(10):1629–35.

250. Lim P, Alker AP, Khim N, Shah NK, Incardona S, Doung S, et al. Pfmdr1 copy number and arteminisin derivatives combination therapy failure in falciparum malaria in Cambodia. Malar J [Internet] 2009 [cited 2025 Jul 16];8(1):11. Available from: https://doi.org/10.1186/1475-2875-8-11

251. Spring MD, Lin JT, Manning JE, Vanachayangkul P, Somethy S, Bun R, et al. Dihydroartemisinin-piperaquine failure associated with a triple mutant including kelch13 C580Y in Cambodia: an observational cohort study. Lancet Infect Dis 2015;15(6):683–91.

252. Quang Bui P, Hong Huynh Q, Thanh Tran D, Thanh Le D, Quang Nguyen T, Van Truong H, et al. Pyronaridine-artesunate Efficacy and Safety in Uncomplicated Plasmodium falciparum Malaria in Areas of Artemisinin-resistant Falciparum in Viet Nam (2017-2018). Clin Infect Dis 2020;70(10):2187–95.

253. A Plasmodium falciparum genetic cross reveals the contributions of pfcrt and plasmepsin II/III to piperaquine drug resistance | mBio [Internet]. [cited 2025 Sep 26];Available from: https://journals.asm.org/doi/10.1128/mbio.00805-24

254. Dong Y, Wang J, Sun A, Deng Y, Chen M, Xu Y, et al. Genetic association between the Pfk13 gene mutation and artemisinin resistance phenotype in Plasmodium falciparum isolates from Yunnan Province, China. Malar J 2018;17(1):478.

255. Norahmad NA, Mohd Abd Razak MR, Abdullah NR, Sastu UR, Imwong M, Muniandy PK, et al. Prevalence of Plasmodium falciparum Molecular Markers of Antimalarial Drug Resistance in a Residual Malaria Focus Area in Sabah, Malaysia. PLoS One 2016;11(10):e0165515.

256. Atroosh WM, Al-Mekhlafi HM, Mahdy MAK, Surin J. The detection of pfcrt and pfmdr1 point mutations as molecular markers of chloroquine drug resistance, Pahang, Malaysia. Malar J 2012;11:251.

257. Yoshida N, Yamauchi M, Morikawa R, Hombhanje F, Mita T. Increase in the proportion of Plasmodium falciparum with kelch13 C580Y mutation and decline in pfcrt and pfmdr1 mutant alleles in Papua New Guinea. Malar J 2021;20(1):410.

258. Huang F, Tang L, Yang H, Zhou S, Liu H, Li J, et al. Molecular epidemiology of drug resistance markers of Plasmodium falciparum in Yunnan Province, China. Malar J 2012;11:243.

259. Mayxay M, Barends M, Brockman A, ... In vitro antimalarial drug susceptibility and pfcrt mutation among fresh Plasmodium falciparum isolates from the Lao PDR (Laos). … American journal of … [Internet] 2007;(Query date: 2025-07-02 12:44:41). Available from: https://pmc.ncbi.nlm.nih.gov/articles/PMC7610946/

260. Huang F, Yan H, Xue JB, Cui YW, Zhou SS, Xia ZG, et al. Molecular surveillance of pfcrt, pfmdr1 and pfk13-propeller mutations in Plasmodium falciparum isolates imported from Africa to China. Malar J 2021;20(1):73.

261. Gresty KJ, Gray KA, Bobogare A, Taleo G, Hii J, Wini L, et al. Genetic mutations in pfcrt and pfmdr1 at the time of artemisinin combination therapy introduction in South Pacific islands of Vanuatu and Solomon Islands. Malar J 2014;13:406.
